# Supplementary material for: Sex ratios and union formation in the historical population of the St. Lawrence Valley
Source: PLoS One. 2022 Jun 8;17(6):e0268039. doi: 10.1371/journal.pone.0268039 (PMC9176776; doi:10.1371/journal.pone.0268039)
Supplement: S1 Table — (PDF) [file pone.0268039.s001.pdf]

Full regression tables from Cox proportional hazards models

1 Women, retrospective localization

Colonial: Hazard ratios from Cox regression models for women's transition into first marriage using the colonial sex ratio from retrospective localization

	I	I sfe	I pfe	I bfe	II	II sfe	II pfe	II bfe	III	III sfe	III pfe	III bfe	IV	IV sfe	IV pfe	IV bfe
SR 50-55%	0.746***	0.709***	0.695***	0.719***	0.265***	0.117***	0.240***	0.253***	0.752***	0.798***	0.701***	0.723***	0.268***	0.224***	0.241***	0.252***
	(0.021)	(0.058)	(0.022)	(0.023)	(0.137)	(0.259)	(0.139)	(0.139)	(0.021)	(0.062)	(0.022)	(0.023)	(0.137)	(0.307)	(0.139)	(0.139)
SR 55-59%	ref.	ref.	ref.	ref.	ref.	ref.	ref.	ref.	ref.	ref.	ref.	ref.	ref.	ref.	ref.	ref.
SR 60-65%	1.980***	1.880***	1.891***	1.873***	1.022	1.173	0.995	1.018	1.959***	1.680***	1.884***	1.868***	1.014	1.127	0.990	1.013
	(0.051)	(0.096)	(0.052)	(0.053)	(0.075)	(0.113)	(0.075)	(0.076)	(0.051)	(0.098)	(0.053)	(0.053)	(0.075)	(0.114)	(0.075)	(0.076)
SR >65%	2.094***	2.447***	2.015***	2.032***	0.724*	0.832	0.724*	0.738+	2.073***	1.997***	2.023***	2.043***	0.718*	0.786	0.721*	0.734+
	(0.086)	(0.168)	(0.088)	(0.088)	(0.160)	(0.262)	(0.161)	(0.161)	(0.086)	(0.170)	(0.088)	(0.088)	(0.160)	(0.262)	(0.161)	(0.161)
decade 1670-79					ref.	ref.	ref.	ref.					ref.	ref.	ref.	ref.
decade 1680-89					0.627***	0.442***	0.639***	0.628***					0.626***	0.487**	0.635***	0.623***
					(0.124)	(0.220)	(0.126)	(0.126)					(0.125)	(0.221)	(0.126)	(0.126)
decade 1690-99					0.377***	0.253***	0.386***	0.387***					0.376***	0.302***	0.382***	0.383***
					(0.139)	(0.242)	(0.141)	(0.140)					(0.139)	(0.246)	(0.141)	(0.140)
decade 1700-09					0.286***	0.173***	0.291***	0.297***					0.287***	0.224***	0.289***	0.293***
					(0.139)	(0.247)	(0.141)	(0.140)					(0.139)	(0.255)	(0.141)	(0.140)
decade 1710-19					0.962	1.163*	1.064*	1.028					0.948+	0.848	1.050+	1.014
					(0.028)	(0.069)	(0.029)	(0.029)					(0.028)	(0.105)	(0.029)	(0.029)
decade 1720-29					0.908***	1.041	0.982	0.957+					0.902***	0.842*	0.975	0.951+
					(0.024)	(0.055)	(0.026)	(0.026)					(0.025)	(0.077)	(0.026)	(0.026)
decade 1730-39					0.987	1.065	1.025	1.014					0.991	0.959	1.029	1.017
					(0.023)	(0.042)	(0.024)	(0.024)					(0.023)	(0.050)	(0.024)	(0.024)
decade 1740-49					NA	NA	NA	NA					NA	NA	NA	NA
					(0.000)	(0.000)	(0.000)	(0.000)					(0.000)	(0.000)	(0.000)	(0.000)
Birth rank									0.982***	0.958***	0.985***	0.983***	0.983***	0.959***	0.986***	0.984***
									(0.003)	(0.006)	(0.003)	(0.003)	(0.003)	(0.008)	(0.003)	(0.003)

	I	I sfe	I pfe	I bfe	II	II sfe	II pfe	II bfe	III	III sfe	III pfe	III bfe	IV	IV sfe	IV pfe	IV bfe
Total # of siblings									0.992*	1.012	0.991**	0.992*	0.992*	1.013	0.992*	0.992*
									(0.003)	(0.016)	(0.003)	(0.003)	(0.003)	(0.016)	(0.003)	(0.003)
# of sisters alive (at age 14)									0.998	1.029	0.998	0.999	1.001	1.031	0.999	1.000
									(0.006)	(0.021)	(0.006)	(0.006)	(0.006)	(0.021)	(0.006)	(0.006)
# of brothers alive (at age 14)									1.008	0.930*	0.992	0.993	1.006	0.929*	0.992	0.991
									(0.009)	(0.036)	(0.009)	(0.009)	(0.009)	(0.036)	(0.009)	(0.009)
Mother alive									ref.	ref.	ref.	ref.	ref.	ref.	ref.	ref.
Mother dead									1.008	1.292***	1.004	1.005	1.018	1.294***	1.013	1.013
									(0.020)	(0.065)	(0.021)	(0.021)	(0.020)	(0.065)	(0.021)	(0.021)
Father alive									ref.	ref.	ref.	ref.	ref.	ref.	ref.	ref.
Father dead									1.103***	1.272***	1.119***	1.115***	1.113***	1.278***	1.125***	1.123***
									(0.019)	(0.047)	(0.019)	(0.019)	(0.019)	(0.048)	(0.019)	(0.019)
Num.Obs.	184195	184094	184195	184195	184195	184094	184195	184195	183857	183857	183857	183857	183857	183857	183857	183857
AIC	282954.1	23224.1	163301.9	170472.4	282782.3	23134.9	163141.7	170330.2	282145.1	23120.7	162711.8	169880.0	281971.3	23055.8	162551.9	169735.7
BIC	282977.1	23247.0	163324.9	170495.4	282851.2	23203.7	163210.6	170399.1	282214.0	23189.6	162780.7	169948.9	282086.1	23170.6	162666.7	169850.5
Log.Lik.	-141474.054	-11609.038	-81647.960	-85233.216	-141382.131	-11558.429	-81561.848	-85156.116	-141063.557	-11551.369	-81346.914	-84930.996	-140970.657	-11512.921	-81260.943	-84852.862
concordance	0.553	0.517	0.564	0.567	0.559	0.535	0.572	0.577	0.571	0.558	0.585	0.586	0.572	0.559	0.588	0.591
n	184195	184094	184195	184195	184195	184094	184195	184195	183857	183857	183857	183857	183857	183857	183857	183857
nevent	15601	15588	15601	15601	15601	15588	15601	15601	15565	15565	15565	15565	15565	15565	15565	15565
p.value.log	0	0	0	0	0	0	0	0	0	0	0	0	0	0	0	0
p.value.sc	0	0	0	0	0	0	0	0	0	0	0	0	0	0	0	0
p.value.wald	0	0	0	0	0	0	0	0	0	0	0	0	0	0	0	0
r.squared.max	0.785	0.119	0.589	0.605	0.785	0.119	0.589	0.605	0.785	0.119	0.589	0.604	0.785	0.119	0.589	0.604
statistic.log	559.942	88.130	588.048	504.490	743.787	189.348	760.272	658.690	663.396	174.696	706.609	621.965	849.195	251.591	878.550	778.233
statistic.sc	722.137	88.581	712.230	601.668	990.256	189.800	957.416	809.226	824.651	174.995	831.733	719.145	1095.027	251.827	1077.457	929.308
statistic.wald	681.450	86.950	679.450	576.650	922.940	183.510	900.390	764.750	783.600	172.340	799.030	694.180	1027.100	244.070	1020.560	884.970
std.error.concordance	0.002	0.002	0.003	0.003	0.003	0.004	0.004	0.004	0.003	0.005	0.004	0.004	0.003	0.005	0.004	0.004

+ p < 0.1, * p < 0.05, ** p < 0.01, *** p < 0.001

Regional: Hazard ratios from Cox regression models for women's transition into first marriage using the regional sex ratio from retrospective localization

	I	I sfe	I pfe	I bfe	II	II sfe	II pfe	II bfe	III	III sfe	III pfe	III bfe	IV	IV sfe	IV pfe	IV bfe
SR <45%	0.703**	1.081	0.852	0.842	0.737**	0.996	0.971	0.917	0.701**	1.037	0.854	0.833	0.737**	0.989	0.965	0.902
	(0.110)	(0.193)	(0.128)	(0.119)	(0.111)	(0.192)	(0.126)	(0.119)	(0.110)	(0.193)	(0.128)	(0.119)	(0.111)	(0.193)	(0.126)	(0.119)
SR 45-50%	0.759***	0.923	0.768***	0.777***	0.820***	0.949	0.960	0.905**	0.745***	0.933	0.765***	0.769***	0.805***	0.945	0.949	0.890**
	(0.034)	(0.068)	(0.038)	(0.036)	(0.034)	(0.069)	(0.041)	(0.038)	(0.034)	(0.069)	(0.038)	(0.037)	(0.035)	(0.070)	(0.041)	(0.038)
SR 50-55%	0.863***	0.960	0.852***	0.863***	0.926***	0.992	0.990	0.954*	0.860***	0.971	0.853***	0.861***	0.922***	0.997	0.988	0.950*
	(0.018)	(0.037)	(0.022)	(0.021)	(0.020)	(0.038)	(0.024)	(0.022)	(0.018)	(0.037)	(0.022)	(0.021)	(0.020)	(0.038)	(0.024)	(0.022)
SR 55-59%	ref.	ref.	ref.	ref.	ref.	ref.	ref.	ref.	ref.	ref.	ref.	ref.	ref.	ref.	ref.	ref.
SR 60-65%	1.460***	1.197*	1.475***	1.410***	0.993	0.929	0.910	0.989	1.440***	1.098	1.466***	1.404***	0.992	0.922	0.913	0.991
	(0.045)	(0.080)	(0.049)	(0.047)	(0.054)	(0.086)	(0.057)	(0.055)	(0.045)	(0.080)	(0.049)	(0.047)	(0.054)	(0.086)	(0.057)	(0.055)
SR >65%	2.588***	1.934***	2.281***	2.484***	1.499***	1.413*	1.173+	1.511***	2.546***	1.739***	2.248***	2.471***	1.495***	1.397*	1.166+	1.508***
	(0.064)	(0.127)	(0.072)	(0.066)	(0.078)	(0.138)	(0.087)	(0.080)	(0.064)	(0.128)	(0.072)	(0.066)	(0.078)	(0.138)	(0.087)	(0.080)
decade 1670-79					ref.	ref.	ref.	ref.					ref.	ref.	ref.	ref.
decade 1680-89					0.900	0.602**	0.827*	0.892					0.903	0.676*	0.823*	0.888
					(0.088)	(0.162)	(0.090)	(0.089)					(0.087)	(0.164)	(0.090)	(0.089)
decade 1690-99					0.577***	0.321***	0.501***	0.584***					0.581***	0.391***	0.498***	0.582***
					(0.094)	(0.185)	(0.098)	(0.096)					(0.094)	(0.192)	(0.098)	(0.096)
decade 1700-09					0.445***	0.212***	0.382***	0.458***					0.449***	0.278***	0.380***	0.455***
					(0.094)	(0.192)	(0.098)	(0.096)					(0.094)	(0.207)	(0.098)	(0.097)
decade 1710-19					0.410***	0.170***	0.333***	0.408***					0.413***	0.239***	0.331***	0.405***
					(0.094)	(0.199)	(0.099)	(0.097)					(0.094)	(0.223)	(0.099)	(0.097)
decade 1720-29					0.396***	0.156***	0.310***	0.390***					0.402***	0.243***	0.311***	0.390***
					(0.093)	(0.204)	(0.099)	(0.096)					(0.093)	(0.240)	(0.099)	(0.096)
decade 1730-39					0.422***	0.154***	0.315***	0.404***					0.433***	0.264***	0.319***	0.408***
					(0.093)	(0.208)	(0.099)	(0.096)					(0.093)	(0.259)	(0.099)	(0.097)
decade 1740-49					0.427***	0.141***	0.310***	0.398***					0.437***	0.268***	0.312***	0.400***
					(0.092)	(0.211)	(0.098)	(0.096)					(0.092)	(0.280)	(0.098)	(0.096)
Birth rank									0.983***	0.951***	0.986***	0.985***	0.983***	0.961***	0.987***	0.985***

	I	I sfe	I pfe	I bfe	II	II sfe	II pfe	II bfe	III	III sfe	III pfe	III bfe	IV	IV sfe	IV pfe	IV bfe
									(0.003)	(0.006)	(0.003)	(0.003)	(0.003)	(0.009)	(0.003)	(0.003)
Total # of siblings									0.990**	1.021	0.986***	0.988***	0.992*	1.021	0.990**	0.992*
									(0.003)	(0.017)	(0.004)	(0.004)	(0.003)	(0.017)	(0.004)	(0.004)
# of sisters alive (at age 14)									0.998	1.050*	1.003	1.002	0.998	1.049*	0.997	0.997
									(0.007)	(0.022)	(0.007)	(0.007)	(0.007)	(0.022)	(0.007)	(0.007)
# of brothers alive (at age 14)									1.001	0.905**	0.989	0.990	1.006	0.902**	0.993	0.993
									(0.009)	(0.038)	(0.009)	(0.009)	(0.009)	(0.038)	(0.009)	(0.009)
Mother alive									ref.	ref.	ref.	ref.	ref.	ref.	ref.	ref.
Mother dead									0.996	1.261***	0.982	0.986	1.013	1.282***	1.004	1.006
									(0.021)	(0.068)	(0.022)	(0.021)	(0.021)	(0.069)	(0.022)	(0.022)
Father alive									ref.	ref.	ref.	ref.	ref.	ref.	ref.	ref.
Father dead									1.105***	1.281***	1.111***	1.107***	1.118***	1.286***	1.129***	1.124***
									(0.020)	(0.050)	(0.020)	(0.020)	(0.020)	(0.050)	(0.020)	(0.020)
Num.Obs.	171081	171048	171081	171081	171081	171048	171081	171081	170842	170842	170842	170842	170842	170842	170842	170842
AIC	258910.8	20787.8	147547.1	154690.0	258632.3	20660.7	147181.3	154439.4	258329.9	20662.0	147122.3	154275.2	258063.5	20588.9	146770.2	154031.3
BIC	258948.7	20825.7	147584.9	154727.9	258723.2	20751.6	147272.2	154530.3	258413.2	20745.3	147205.6	154358.5	258199.8	20725.2	146906.5	154167.6
Log.Lik.	-129450.422	-10388.891	-73768.525	-77340.017	-129304.153	-10318.331	-73578.633	-77207.716	-129153.953	-10319.988	-73550.168	-77126.591	-129013.763	-10276.462	-73367.097	-76997.656
concordance	0.547	0.514	0.555	0.560	0.564	0.542	0.573	0.578	0.561	0.557	0.575	0.576	0.575	0.556	0.589	0.591
n	171081	171048	171081	171081	171081	171048	171081	171081	170842	170842	170842	170842	170842	170842	170842	170842
nevent	14389	14386	14389	14389	14389	14386	14389	14389	14365	14365	14365	14365	14365	14365	14365	14365
p.value.log	0	0	0	0	0	0	0	0	0	0	0	0	0	0	0	0
p.value.sc	0	0	0	0	0	0	0	0	0	0	0	0	0	0	0	0
p.value.wald	0	0	0	0	0	0	0	0	0	0	0	0	0	0	0	0
r.squared.max	0.780	0.115	0.579	0.596	0.780	0.115	0.579	0.596	0.780	0.115	0.578	0.596	0.780	0.115	0.578	0.596
statistic.log	416.570	31.860	295.574	336.501	709.109	172.979	675.358	601.103	531.760	145.053	423.643	455.907	812.140	232.105	789.785	713.777
statistic.sc	531.217	32.722	354.402	421.626	937.854	173.486	843.417	755.068	645.449	145.390	482.891	541.550	1040.454	232.197	959.594	868.620
statistic.wald	500.610	32.090	341.370	399.820	875.320	167.610	795.500	710.010	614.500	143.700	469.700	519.510	977.410	224.930	911.760	823.380
std.error.concordance	0.003	0.004	0.004	0.004	0.003	0.005	0.004	0.004	0.003	0.006	0.004	0.004	0.003	0.006	0.004	0.004

+ p < 0.1, * p < 0.05, ** p < 0.01, *** p < 0.001

Parish-level: Hazard ratios from Cox regression models forwomen's transition into first marriage using the parish-level sex ratio from retrospective localization

	I	I sfe	I pfe	I bfe	II	II sfe	II pfe	II bfe	III	III sfe	III pfe	III bfe	IV	IV sfe	IV pfe	IV bfe
SR <45%	0.799***	1.007	0.838***	0.861**	0.824***	0.972	0.875*	0.902*	0.798***	0.960	0.841**	0.861**	0.825***	0.963	0.879*	0.902*
	(0.047)	(0.078)	(0.053)	(0.050)	(0.047)	(0.079)	(0.053)	(0.050)	(0.047)	(0.078)	(0.053)	(0.050)	(0.047)	(0.079)	(0.054)	(0.050)
SR 45-50%	0.786***	0.945	0.813***	0.830***	0.821***	0.957	0.945+	0.916**	0.775***	0.933	0.811***	0.822***	0.812***	0.955	0.940*	0.906***
	(0.024)	(0.047)	(0.029)	(0.027)	(0.025)	(0.048)	(0.030)	(0.027)	(0.024)	(0.048)	(0.029)	(0.027)	(0.025)	(0.049)	(0.030)	(0.028)
SR 50-55%	0.882***	0.934+	0.906***	0.914***	0.905***	0.942+	0.966	0.951*	0.878***	0.927*	0.905***	0.911***	0.902***	0.943	0.965	0.949*
	(0.021)	(0.036)	(0.023)	(0.022)	(0.021)	(0.036)	(0.023)	(0.022)	(0.021)	(0.036)	(0.023)	(0.022)	(0.021)	(0.036)	(0.023)	(0.022)
SR 55-59%	ref.	ref.	ref.	ref.	ref.	ref.	ref.	ref.	ref.	ref.	ref.	ref.	ref.	ref.	ref.	ref.
SR 60-65%	1.262***	1.144*	1.253***	1.252***	1.124***	1.066	1.029	1.116**	1.256***	1.114+	1.245***	1.247***	1.124***	1.071	1.027	1.115**
	(0.032)	(0.056)	(0.035)	(0.034)	(0.034)	(0.057)	(0.037)	(0.035)	(0.033)	(0.056)	(0.035)	(0.034)	(0.034)	(0.057)	(0.037)	(0.035)
SR >65%	2.495***	2.011***	1.944***	2.493***	1.950***	1.779***	1.262***	2.002***	2.465***	1.899***	1.905***	2.474***	1.947***	1.767***	1.247***	1.995***
	(0.044)	(0.083)	(0.059)	(0.046)	(0.048)	(0.086)	(0.063)	(0.049)	(0.044)	(0.084)	(0.059)	(0.046)	(0.048)	(0.086)	(0.063)	(0.049)
decade 1670-79					ref.	ref.	ref.	ref.					ref.	ref.	ref.	ref.
decade 1680-89					0.962	0.639**	0.834*	0.961					0.968	0.713*	0.830*	0.959
					(0.083)	(0.155)	(0.085)	(0.084)					(0.083)	(0.157)	(0.085)	(0.084)
decade 1690-99					0.680***	0.371***	0.532***	0.690***					0.686***	0.450***	0.528***	0.687***
					(0.082)	(0.173)	(0.086)	(0.084)					(0.082)	(0.181)	(0.086)	(0.084)
decade 1700-09					0.529***	0.245***	0.408***	0.541***					0.534***	0.317***	0.404***	0.538***
					(0.082)	(0.181)	(0.086)	(0.084)					(0.082)	(0.198)	(0.086)	(0.085)
decade 1710-19					0.499***	0.199***	0.361***	0.491***					0.502***	0.277***	0.358***	0.486***
					(0.081)	(0.189)	(0.086)	(0.084)					(0.081)	(0.215)	(0.086)	(0.084)
decade 1720-29					0.481***	0.182***	0.338***	0.471***					0.487***	0.279***	0.336***	0.470***
					(0.080)	(0.193)	(0.086)	(0.083)					(0.080)	(0.233)	(0.086)	(0.084)
decade 1730-39					0.506***	0.180***	0.342***	0.487***					0.519***	0.303***	0.344***	0.491***
					(0.079)	(0.197)	(0.085)	(0.083)					(0.079)	(0.253)	(0.086)	(0.083)
decade 1740-49					0.515***	0.163***	0.336***	0.479***					0.525***	0.303***	0.337***	0.481***
					(0.079)	(0.200)	(0.085)	(0.083)					(0.079)	(0.274)	(0.085)	(0.083)
Birth rank									0.981***	0.951***	0.984***	0.984***	0.981***	0.962***	0.987***	0.984***

	I	I sfe	I pfe	I bfe	II	II sfe	II pfe	II bfe	III	III sfe	III pfe	III bfe	IV	IV sfe	IV pfe	IV bfe
									(0.003)	(0.006)	(0.003)	(0.003)	(0.003)	(0.009)	(0.003)	(0.003)
Total # of siblings									0.993*	1.024	0.987***	0.990**	0.995	1.023	0.991*	0.993+
									(0.003)	(0.017)	(0.004)	(0.004)	(0.003)	(0.018)	(0.004)	(0.004)
# of sisters alive (at age 14)									1	1.050*	1.004	1.003	1	1.049*	0.998	0.999
									(0.007)	(0.022)	(0.007)	(0.007)	(0.007)	(0.022)	(0.007)	(0.007)
# of brothers alive (at age 14)									0.995	0.900**	0.989	0.988	1	0.898**	0.992	0.990
									(0.009)	(0.038)	(0.009)	(0.009)	(0.009)	(0.038)	(0.009)	(0.009)
Mother alive									ref.	ref.	ref.	ref.	ref.	ref.	ref.	ref.
Mother dead									0.993	1.249**	0.977	0.982	1.017	1.273***	1.005	1.006
									(0.021)	(0.068)	(0.021)	(0.021)	(0.021)	(0.068)	(0.022)	(0.021)
Father alive									ref.	ref.	ref.	ref.	ref.	ref.	ref.	ref.
Father dead									1.111***	1.283***	1.111***	1.114***	1.127***	1.287***	1.128***	1.130***
									(0.019)	(0.049)	(0.020)	(0.020)	(0.020)	(0.050)	(0.020)	(0.020)
Num.Obs.	171401	171368	171401	171401	171401	171368	171401	171401	171162	171162	171162	171162	171162	171162	171162	171162
AIC	260627.5	20933.3	148309.1	155708.9	260351.9	20814.3	147879.3	155457.4	260046.6	20805.8	147888.3	155299.3	259784.6	20743.3	147472.7	155053.7
BIC	260665.4	20971.2	148347.0	155746.8	260442.9	20905.3	147970.3	155548.4	260130.0	20889.2	147971.7	155382.7	259921.0	20879.7	147609.1	155190.1
Log.Lik.	-130308.752	-10461.669	-74149.539	-77849.470	-130163.955	-10395.172	-73927.645	-77716.697	-130012.316	-10391.893	-73933.152	-77638.671	-129874.284	-10353.634	-73718.344	-77508.825
concordance	0.551	0.518	0.552	0.562	0.573	0.544	0.573	0.583	0.565	0.562	0.573	0.578	0.583	0.561	0.590	0.597
n	171401	171368	171401	171401	171401	171368	171401	171401	171162	171162	171162	171162	171162	171162	171162	171162
nevent	14493	14490	14493	14493	14493	14490	14493	14493	14469	14469	14469	14469	14469	14469	14469	14469
p.value.log	0	0	0	0	0	0	0	0	0	0	0	0	0	0	0	0
p.value.sc	0	0	0	0	0	0	0	0	0	0	0	0	0	0	0	0
p.value.wald	0	0	0	0	0	0	0	0	0	0	0	0	0	0	0	0
r.squared.max	0.782	0.115	0.580	0.598	0.782	0.115	0.580	0.598	0.782	0.115	0.579	0.598	0.782	0.115	0.579	0.598
statistic.log	661.358	86.598	262.938	526.274	950.952	219.592	706.725	791.819	776.182	201.536	387.101	640.198	1052.247	278.054	816.718	899.888
statistic.sc	857.880	90.021	296.971	677.025	1279.358	221.717	883.957	1026.571	970.854	204.158	421.521	790.557	1379.195	279.672	995.551	1134.868
statistic.wald	803	87.640	290.910	638.290	1190.360	213	832.510	962.500	916.130	199.870	415.440	751.970	1289.830	269.230	944.450	1070.840
std.error.concordance	0.003	0.005	0.004	0.004	0.003	0.005	0.004	0.004	0.003	0.006	0.004	0.004	0.003	0.006	0.004	0.004

+ p < 0.1, * p < 0.05, ** p < 0.01, *** p < 0.001

2 Men, retrospective localization

Colonial: Hazard ratios from Cox regression models for men's transition into first marriage using the colonial sex ratio from retrospective localization

	I	I sfe	I pfe	I bfe	II	II sfe	II pfe	II bfe	III	III sfe	III pfe	III bfe	IV	IV sfe	IV pfe	IV bfe
SR 50-55%	1.115***	1.232**	1.038	1.074**	0.901	1.058	0.730	0.809	1.120***	1.238**	1.043	1.079**	0.904	1.284	0.724	0.807
	(0.025)	(0.066)	(0.026)	(0.027)	(0.220)	(0.340)	(0.222)	(0.222)	(0.025)	(0.070)	(0.026)	(0.027)	(0.221)	(0.388)	(0.222)	(0.222)
SR 55-59%	ref.	ref.	ref.	ref.	ref.	ref.	ref.	ref.	ref.	ref.	ref.	ref.	ref.	ref.	ref.	ref.
SR 60-65%	0.914	0.804+	0.944	0.983	0.763*	0.724*	0.756**	0.805*	0.920	0.793+	0.957	0.992	0.765*	0.713*	0.760*	0.809*
	(0.070)	(0.120)	(0.071)	(0.071)	(0.106)	(0.156)	(0.107)	(0.107)	(0.070)	(0.122)	(0.071)	(0.071)	(0.106)	(0.156)	(0.107)	(0.107)
SR >65%	0.622**	0.418***	0.647**	0.665**	0.500**	0.379**	0.478**	0.521*	0.626**	0.408***	0.657**	0.672**	0.502**	0.376**	0.482**	0.524*
	(0.149)	(0.253)	(0.150)	(0.151)	(0.265)	(0.371)	(0.265)	(0.265)	(0.149)	(0.255)	(0.150)	(0.151)	(0.265)	(0.372)	(0.265)	(0.265)
decade 1670-79					ref.	ref.	ref.	ref.					ref.	ref.	ref.	ref.
decade 1680-89					0.958	1.005	0.912	0.947					0.960	1.041	0.912	0.947
					(0.205)	(0.291)	(0.205)	(0.205)					(0.205)	(0.292)	(0.205)	(0.205)
decade 1690-99					0.903	0.900	0.836	0.873					0.902	0.957	0.831	0.870
					(0.223)	(0.323)	(0.223)	(0.223)					(0.223)	(0.327)	(0.223)	(0.223)
decade 1700-09					0.720	0.765	0.643*	0.690+					0.717	0.832	0.635*	0.683+
					(0.222)	(0.328)	(0.223)	(0.223)					(0.222)	(0.336)	(0.223)	(0.223)
decade 1710-19					0.968	0.905	1.058+	1.031					0.963	0.829	1.058+	1.029
					(0.031)	(0.078)	(0.032)	(0.032)					(0.031)	(0.118)	(0.032)	(0.032)
decade 1720-29					0.991	0.979	1.062*	1.031					0.988	0.922	1.065*	1.030
					(0.027)	(0.060)	(0.028)	(0.028)					(0.027)	(0.086)	(0.028)	(0.028)
decade 1730-39					1.005	0.992	1.043	1.027					1.003	0.960	1.043	1.026
					(0.025)	(0.046)	(0.026)	(0.026)					(0.025)	(0.056)	(0.026)	(0.026)
decade 1740-49					NA	NA	NA	NA					NA	NA	NA	NA
					(0)	(0)	(0)	(0)					(0)	(0)	(0)	(0)
Birth rank									1.006+	0.990	1.007*	1.006+	1.007*	0.984+	1.009*	1.006+
									(0.003)	(0.007)	(0.003)	(0.003)	(0.003)	(0.009)	(0.003)	(0.003)
Total # of siblings									0.986***	0.989	0.988**	0.988**	0.986***	0.990	0.988**	0.988**

	I	I sfe	I pfe	I bfe	II	II sfe	II pfe	II bfe	III	III sfe	III pfe	III bfe	IV	IV sfe	IV pfe	IV bfe
									(0.004)	(0.019)	(0.004)	(0.004)	(0.004)	(0.019)	(0.004)	(0.004)
# of sisters alive (at age 14)									0.999	0.981	0.991	0.993	1	0.984	0.990	0.993
									(0.007)	(0.032)	(0.007)	(0.007)	(0.007)	(0.032)	(0.007)	(0.007)
# of brothers alive (at age 14)									1.018+	0.998	1.005	1.006	1.018+	0.994	1.004	1.006
									(0.009)	(0.040)	(0.010)	(0.009)	(0.009)	(0.040)	(0.010)	(0.009)
Mother alive									ref.	ref.	ref.	ref.	ref.	ref.	ref.	ref.
Mother dead									1.028	1.075	1.013	1.017	1.032	1.069	1.017	1.021
									(0.022)	(0.071)	(0.022)	(0.022)	(0.022)	(0.071)	(0.022)	(0.022)
Father alive									ref.	ref.	ref.	ref.	ref.	ref.	ref.	ref.
Father dead									0.997	1.057	1.004	0.999	1.001	1.067	1.006	1.002
									(0.020)	(0.052)	(0.020)	(0.020)	(0.020)	(0.052)	(0.020)	(0.020)
Num.Obs.	228824	228680	228824	228824	228824	228680	228824	228824	228543	228543	228543	228543	228543	228543	228543	228543
AIC	216314.6	17336.6	121811.1	128428.2	216297.9	17340.7	121780.4	128409.8	216025.2	17329.1	121599.6	128214.9	216007.4	17332.2	121567.0	128195.4
BIC	216336.8	17358.9	121833.3	128450.5	216364.7	17407.4	121847.2	128476.5	216091.9	17395.9	121666.3	128281.6	216118.6	17443.4	121678.2	128306.6
Log.Lik.	-108154.280	-8665.325	-60902.529	-64211.106	-108139.973	-8661.351	-60881.217	-64195.878	-108003.581	-8655.567	-60790.816	-64098.432	-107988.677	-8651.110	-60768.520	-64082.694
concordance	0.511	0.510	0.512	0.514	0.516	0.516	0.521	0.516	0.524	0.521	0.523	0.525	0.529	0.529	0.531	0.530
n	228824	228680	228824	228824	228824	228680	228824	228824	228543	228543	228543	228543	228543	228543	228543	228543
nevent	12263	12256	12263	12263	12263	12256	12263	12263	12249	12249	12249	12249	12249	12249	12249	12249
p.value.log	0	0	0.002	0	0	0	0	0	0	0	0	0	0	0.001	0	0
p.value.sc	0	0	0.004	0.001	0	0	0	0	0	0.001	0	0	0	0.001	0	0
p.value.wald	0	0	0.005	0.001	0	0	0	0	0	0.001	0	0	0	0.001	0	0
r.squared.max	0.612	0.073	0.413	0.430	0.612	0.073	0.413	0.430	0.611	0.073	0.413	0.429	0.611	0.073	0.413	0.429
statistic.log	44.934	23.680	14.563	18.316	73.547	31.628	57.186	48.772	68.103	29.673	48.787	44.971	97.911	38.586	93.378	76.448
statistic.sc	41.511	22.973	13.174	17.001	68.223	30.866	55.524	46.583	64.581	29.066	47.178	43.562	92.464	37.935	91.483	74.134
statistic.wald	41.070	22.600	13.010	16.840	67.600	30.440	55.200	46.310	64.140	28.660	47.010	43.400	91.840	37.460	91.160	73.850
std.error.concordance	0.002	0.002	0.003	0.003	0.003	0.005	0.004	0.004	0.003	0.006	0.004	0.004	0.003	0.006	0.004	0.004

+ p < 0.1, * p < 0.05, ** p < 0.01, *** p < 0.001

Regional: Hazard ratios from Cox regression models for men's transition into first marriage using the regional sex ratio from retrospective localization

	I	I sfe	I pfe	I bfe	II	II sfe	II pfe	II bfe	III	III sfe	III pfe	III bfe	IV	IV sfe	IV pfe	IV bfe
SR <45%	1.163	1.230	0.924	1.447***	1.139	1.212	0.925	1.433**	1.178	1.219	0.928	1.455***	1.153	1.203	0.927	1.439**
	(0.103)	(0.198)	(0.159)	(0.111)	(0.104)	(0.200)	(0.160)	(0.112)	(0.103)	(0.199)	(0.159)	(0.111)	(0.104)	(0.200)	(0.160)	(0.112)
SR 45-50%	0.894**	1.029	1.014	0.979	0.869***	0.998	1.037	0.972	0.894**	1.025	1.014	0.974	0.867***	0.998	1.037	0.964
	(0.039)	(0.081)	(0.045)	(0.042)	(0.040)	(0.082)	(0.047)	(0.044)	(0.039)	(0.081)	(0.045)	(0.042)	(0.040)	(0.082)	(0.047)	(0.044)
SR 50-55%	1.003	1.041	1.027	1.015	0.985	1.022	1.039	1.010	1.002	1.038	1.024	1.011	0.984	1.023	1.035	1.005
	(0.021)	(0.041)	(0.025)	(0.023)	(0.022)	(0.042)	(0.027)	(0.025)	(0.021)	(0.041)	(0.025)	(0.023)	(0.022)	(0.042)	(0.027)	(0.025)
SR 55-59%	ref.	ref.	ref.	ref.	ref.	ref.	ref.	ref.	ref.	ref.	ref.	ref.	ref.	ref.	ref.	ref.
SR 60-65%	0.812***	0.891	0.870*	0.877*	0.845*	0.920	0.825**	0.872*	0.812***	0.893	0.875*	0.879*	0.845*	0.915	0.828**	0.874*
	(0.057)	(0.095)	(0.060)	(0.059)	(0.067)	(0.102)	(0.070)	(0.068)	(0.057)	(0.096)	(0.060)	(0.059)	(0.067)	(0.102)	(0.070)	(0.068)
SR >65%	0.748**	0.808	0.884	0.875	0.836	0.891	0.872	0.922	0.745**	0.808	0.884	0.873	0.831+	0.881	0.867	0.918
	(0.093)	(0.163)	(0.097)	(0.095)	(0.110)	(0.175)	(0.112)	(0.110)	(0.094)	(0.164)	(0.097)	(0.095)	(0.110)	(0.175)	(0.112)	(0.110)
decade 1670-79					ref.	ref.	ref.	ref.					ref.	ref.	ref.	ref.
decade 1680-89					1.325+	1.474+	1.293+	1.340*					1.327*	1.506+	1.284+	1.337*
					(0.144)	(0.229)	(0.145)	(0.145)					(0.144)	(0.230)	(0.145)	(0.145)
decade 1690-99					1.399*	1.584+	1.317+	1.385*					1.394*	1.654+	1.296+	1.376*
					(0.149)	(0.252)	(0.151)	(0.150)					(0.149)	(0.258)	(0.151)	(0.150)
decade 1700-09					1.104	1.244	0.993	1.074					1.098	1.314	0.970	1.062
					(0.149)	(0.261)	(0.151)	(0.150)					(0.149)	(0.274)	(0.151)	(0.150)
decade 1710-19					1.326+	1.581+	1.138	1.270					1.321+	1.684+	1.116	1.260
					(0.149)	(0.269)	(0.152)	(0.151)					(0.149)	(0.290)	(0.152)	(0.151)
decade 1720-29					1.367*	1.698+	1.140	1.269					1.362*	1.844*	1.121	1.260
					(0.148)	(0.274)	(0.152)	(0.151)					(0.148)	(0.308)	(0.152)	(0.151)
decade 1730-39					1.364*	1.679+	1.094	1.248					1.365*	1.853+	1.075	1.244
					(0.148)	(0.277)	(0.152)	(0.151)					(0.148)	(0.326)	(0.152)	(0.151)
decade 1740-49					1.382*	1.689+	1.068	1.243					1.385*	1.908+	1.049	1.239
					(0.148)	(0.280)	(0.152)	(0.151)					(0.148)	(0.348)	(0.152)	(0.151)
Birth rank									1.006	0.991	1.007*	1.005	1.006	0.983+	1.009*	1.005

	I	I sfe	I pfe	I bfe	II	II sfe	II pfe	II bfe	III	III sfe	III pfe	III bfe	IV	IV sfe	IV pfe	IV bfe
									(0.004)	(0.007)	(0.004)	(0.004)	(0.004)	(0.010)	(0.004)	(0.004)
Total # of siblings									0.987***	0.999	0.987**	0.988**	0.986***	0.999	0.988**	0.988**
									(0.004)	(0.020)	(0.004)	(0.004)	(0.004)	(0.020)	(0.004)	(0.004)
# of sisters alive (at age 14)									0.997	0.968	0.987+	0.990	0.998	0.970	0.987+	0.990
									(0.007)	(0.035)	(0.008)	(0.007)	(0.007)	(0.035)	(0.008)	(0.007)
# of brothers alive (at age 14)									1.020*	0.994	1.005	1.009	1.018+	0.992	1.005	1.009
									(0.010)	(0.043)	(0.010)	(0.010)	(0.010)	(0.043)	(0.010)	(0.010)
Mother alive									ref.	ref.	ref.	ref.	ref.	ref.	ref.	ref.
Mother dead									1.036	1.071	1.012	1.024	1.035	1.063	1.016	1.026
									(0.023)	(0.076)	(0.023)	(0.023)	(0.023)	(0.076)	(0.023)	(0.023)
Father alive									ref.	ref.	ref.	ref.	ref.	ref.	ref.	ref.
Father dead									1.006	1.093	1.009	1.008	1.008	1.095	1.012	1.011
									(0.021)	(0.055)	(0.021)	(0.021)	(0.021)	(0.056)	(0.021)	(0.021)
Num.Obs.	209934	209922	209934	209934	209934	209922	209934	209934	209785	209785	209785	209785	209785	209785	209785	209785
AIC	195411.9	15082.1	107778.3	114886.8	195383.0	15075.8	107750.2	114868.6	195244.2	15071.7	107652.8	114763.9	195213.1	15065.4	107623.3	114744.4
BIC	195448.5	15118.7	107814.9	114923.4	195470.9	15163.7	107838.1	114956.4	195324.7	15152.2	107733.4	114844.4	195344.9	15197.2	107755.1	114876.2
Log.Lik.	-97700.951	-7536.063	-53884.147	-57438.394	-97679.518	-7525.912	-53863.124	-57422.282	-97611.099	-7524.853	-53815.417	-57370.927	-97588.530	-7514.693	-53793.637	-57354.214
concordance	0.513	0.506	0.510	0.511	0.516	0.516	0.523	0.521	0.524	0.517	0.523	0.525	0.529	0.526	0.530	0.530
n	209934	209922	209934	209934	209934	209922	209934	209934	209785	209785	209785	209785	209785	209785	209785	209785
nevent	11188	11187	11188	11188	11188	11187	11188	11188	11180	11180	11180	11180	11180	11180	11180	11180
p.value.log	0	0.403	0.065	0.001	0	0.013	0	0	0	0.233	0	0	0	0.011	0	0
p.value.sc	0	0.404	0.073	0.001	0	0.014	0	0	0	0.231	0	0	0	0.012	0	0
p.value.wald	0	0.405	0.073	0.001	0	0.015	0	0	0	0.232	0	0	0	0.013	0	0
r.squared.max	0.606	0.069	0.402	0.421	0.606	0.069	0.402	0.421	0.606	0.069	0.401	0.421	0.606	0.069	0.401	0.421
statistic.log	36.094	5.111	10.406	19.766	78.960	25.413	52.453	51.990	58.700	14.008	49.907	49.766	103.838	34.328	93.468	83.191
statistic.sc	34.418	5.099	10.089	20.667	74.812	25.110	52.068	51.723	56.921	14.040	49.336	50.580	99.586	34.125	92.862	82.833
statistic.wald	34.280	5.090	10.070	20.520	74.380	24.970	51.930	51.430	56.780	14.010	49.310	50.420	99.150	33.920	92.710	82.530
std.error.concordance	0.003	0.004	0.004	0.004	0.003	0.006	0.004	0.004	0.003	0.007	0.005	0.004	0.003	0.007	0.005	0.004

+ p < 0.1, * p < 0.05, ** p < 0.01, *** p < 0.001

Parish-level: Hazard ratios from Cox regression models for men's transition into first marriage using the parish-level sex ratio from retrospective localization

	I	I sfe	I pfe	I bfe	II	II sfe	II pfe	II bfe	III	III sfe	III pfe	III bfe	IV	IV sfe	IV pfe	IV bfe
SR <45%	1.478***	1.402***	1.398***	1.560***	1.477***	1.398***	1.376***	1.560***	1.478***	1.398***	1.396***	1.561***	1.478***	1.393***	1.373***	1.560***
	(0.052)	(0.092)	(0.071)	(0.053)	(0.052)	(0.093)	(0.071)	(0.054)	(0.052)	(0.092)	(0.071)	(0.053)	(0.052)	(0.093)	(0.071)	(0.054)
SR 45-50%	0.880***	1.024	1.089*	0.982	0.869***	1.004	1.121**	0.980	0.879***	1.020	1.085*	0.975	0.868***	1.003	1.116**	0.972
	(0.029)	(0.060)	(0.035)	(0.032)	(0.030)	(0.061)	(0.037)	(0.033)	(0.030)	(0.060)	(0.035)	(0.032)	(0.030)	(0.061)	(0.037)	(0.033)
SR 50-55%	0.945*	1.070	1.060*	1.015	0.945*	1.065	1.073**	1.019	0.946*	1.070	1.058*	1.013	0.946*	1.067	1.071**	1.016
	(0.023)	(0.042)	(0.026)	(0.025)	(0.023)	(0.042)	(0.027)	(0.025)	(0.023)	(0.042)	(0.026)	(0.025)	(0.024)	(0.042)	(0.027)	(0.025)
SR 55-59%	ref.	ref.	ref.	ref.	ref.	ref.	ref.	ref.	ref.	ref.	ref.	ref.	ref.	ref.	ref.	ref.
SR 60-65%	0.911**	0.927	0.918*	0.935*	0.931*	0.940	0.904**	0.941+	0.914**	0.930	0.924*	0.940+	0.935*	0.941	0.911**	0.947
	(0.032)	(0.052)	(0.034)	(0.033)	(0.033)	(0.053)	(0.035)	(0.034)	(0.032)	(0.052)	(0.034)	(0.033)	(0.033)	(0.053)	(0.035)	(0.034)
SR >65%	0.773***	0.850+	0.806***	0.862**	0.826***	0.875	0.766***	0.875*	0.775***	0.857	0.811***	0.866**	0.830**	0.877	0.772***	0.881*
	(0.053)	(0.095)	(0.060)	(0.055)	(0.057)	(0.098)	(0.065)	(0.059)	(0.053)	(0.096)	(0.060)	(0.055)	(0.057)	(0.098)	(0.065)	(0.059)
decade 1670-79					ref.	ref.	ref.	ref.					ref.	ref.	ref.	ref.
decade 1680-89					1.298+	1.393	1.203	1.291+					1.302+	1.416	1.201	1.291+
					(0.139)	(0.225)	(0.140)	(0.140)					(0.139)	(0.226)	(0.140)	(0.140)
decade 1690-99					1.450**	1.508+	1.257+	1.379*					1.452**	1.555+	1.247	1.376*
					(0.135)	(0.239)	(0.137)	(0.136)					(0.135)	(0.246)	(0.137)	(0.136)
decade 1700-09					1.151	1.192	0.932	1.076					1.149	1.231	0.919	1.070
					(0.134)	(0.249)	(0.137)	(0.137)					(0.134)	(0.264)	(0.137)	(0.137)
decade 1710-19					1.400*	1.505	1.057	1.274+					1.401*	1.552	1.049	1.273+
					(0.133)	(0.256)	(0.137)	(0.136)					(0.133)	(0.281)	(0.137)	(0.137)
decade 1720-29					1.399*	1.550+	1.035	1.246					1.402*	1.612	1.029	1.245
					(0.133)	(0.262)	(0.137)	(0.136)					(0.133)	(0.299)	(0.137)	(0.136)
decade 1730-39					1.404*	1.532	1.007	1.238					1.411**	1.602	1	1.241
					(0.132)	(0.265)	(0.137)	(0.136)					(0.133)	(0.318)	(0.137)	(0.136)
decade 1740-49					1.423**	1.558+	0.980	1.229					1.432**	1.646	0.973	1.233
					(0.132)	(0.268)	(0.137)	(0.136)					(0.132)	(0.341)	(0.137)	(0.136)
Birth rank									1.005	0.992	1.006+	1.004	1.005	0.987	1.008*	1.005

	I	I sfe	I pfe	I bfe	II	II sfe	II pfe	II bfe	III	III sfe	III pfe	III bfe	IV	IV sfe	IV pfe	IV bfe
									(0.004)	(0.007)	(0.004)	(0.004)	(0.004)	(0.010)	(0.004)	(0.004)
Total # of siblings									0.989**	0.996	0.988**	0.990**	0.988**	0.996	0.989**	0.990**
									(0.004)	(0.020)	(0.004)	(0.004)	(0.004)	(0.020)	(0.004)	(0.004)
# of sisters alive (at age 14)									0.995	0.978	0.989	0.989	0.996	0.978	0.988	0.989
									(0.007)	(0.035)	(0.008)	(0.007)	(0.007)	(0.035)	(0.008)	(0.007)
# of brothers alive (at age 14)									1.018+	0.991	1.003	1.008	1.017+	0.990	1.003	1.007
									(0.010)	(0.043)	(0.010)	(0.010)	(0.010)	(0.043)	(0.010)	(0.010)
Mother alive									ref.	ref.	ref.	ref.	ref.	ref.	ref.	ref.
Mother dead									1.032	1.099	1.005	1.015	1.030	1.095	1.011	1.016
									(0.023)	(0.075)	(0.023)	(0.023)	(0.023)	(0.076)	(0.023)	(0.023)
Father alive									ref.	ref.	ref.	ref.	ref.	ref.	ref.	ref.
Father dead									1.007	1.085	1.004	1.006	1.009	1.085	1.007	1.008
									(0.021)	(0.055)	(0.021)	(0.021)	(0.021)	(0.056)	(0.021)	(0.021)
Num.Obs.	210805	210793	210805	210805	210805	210793	210805	210805	210656	210656	210656	210656	210656	210656	210656	210656
AIC	195810.4	15131.8	108179.1	115126.4	195781.7	15128.4	108146.7	115110.7	195647.2	15122.4	108060.1	115008.4	195616.5	15119.3	108027.0	114991.6
BIC	195847.0	15168.4	108215.7	115163.0	195869.6	15216.3	108234.6	115198.6	195727.7	15203.0	108140.7	115088.9	195748.3	15251.2	108158.8	115123.5
Log.Lik.	-97900.213	-7560.905	-54084.540	-57558.200	-97878.847	-7552.224	-54061.367	-57543.330	-97812.576	-7550.207	-54019.064	-57493.192	-97790.241	-7541.662	-53995.491	-57477.822
concordance	0.521	0.520	0.512	0.517	0.527	0.526	0.523	0.525	0.530	0.525	0.524	0.529	0.534	0.533	0.532	0.534
n	210805	210793	210805	210805	210805	210793	210805	210805	210656	210656	210656	210656	210656	210656	210656	210656
nevent	11210	11209	11210	11210	11210	11209	11210	11210	11202	11202	11202	11202	11202	11202	11202	11202
p.value.log	0	0	0	0	0	0	0	0	0	0.001	0	0	0	0	0	0
p.value.sc	0	0	0	0	0	0	0	0	0	0.001	0	0	0	0	0	0
p.value.wald	0	0	0	0	0	0	0	0	0	0.001	0	0	0	0	0	0
r.squared.max	0.605	0.069	0.402	0.421	0.605	0.069	0.402	0.421	0.605	0.069	0.401	0.421	0.605	0.069	0.401	0.421
statistic.log	110.325	22.851	55.426	83.034	153.058	40.212	101.772	112.774	128.473	30.722	88.417	108.170	173.143	47.814	135.563	138.910
statistic.sc	120.601	23.106	55.935	93.316	160.712	40.205	103.087	122.021	138.647	31.020	88.771	118.373	180.723	47.872	136.644	148.074
statistic.wald	118.980	22.980	55.700	91.920	158.790	39.920	102.790	120.480	137.020	30.860	88.520	116.960	178.790	47.520	136.330	146.510
std.error.concordance	0.003	0.005	0.004	0.004	0.003	0.006	0.004	0.004	0.003	0.007	0.004	0.004	0.003	0.007	0.005	0.004

+ p < 0.1, * p < 0.05, ** p < 0.01, *** p < 0.001

3 Women, prospective localization

Colonial: Hazard ratios from Cox regression models for women's transition into first marriage using the colonial sex ratio from prospective localization

	I	I sfe	I pfe	I bfe	II	II sfe	II pfe	II bfe	III	III sfe	III pfe	III bfe	IV	IV sfe	IV pfe	IV bfe
SR 55-59%	ref.	ref.	ref.	ref.	ref.	ref.	ref.	ref.	ref.	ref.	ref.	ref.	ref.	ref.	ref.	ref.
SR 60-65%	1.303***	1.512***	1.415***	1.371***	1.198**	1.349**	1.222**	1.210**	1.293***	1.377***	1.402***	1.364***	1.195*	1.303**	1.216**	1.206**
	(0.025)	(0.060)	(0.025)	(0.026)	(0.070)	(0.097)	(0.070)	(0.070)	(0.025)	(0.063)	(0.025)	(0.026)	(0.070)	(0.098)	(0.070)	(0.070)
SR >65%	2.202***	2.658***	2.401***	2.203***	1.121	1.483**	1.152	1.089	2.170***	2.183***	2.368***	2.190***	1.111	1.352*	1.139	1.080
	(0.031)	(0.091)	(0.032)	(0.034)	(0.100)	(0.141)	(0.101)	(0.101)	(0.031)	(0.099)	(0.033)	(0.035)	(0.101)	(0.144)	(0.101)	(0.101)
decade 1670-79					ref.	ref.	ref.	ref.					ref.	ref.	ref.	ref.
decade 1680-89					0.783**	0.530***	0.752***	0.773**					0.785**	0.595***	0.747***	0.768***
					(0.077)	(0.142)	(0.078)	(0.078)					(0.077)	(0.145)	(0.078)	(0.078)
decade 1690-99					0.444***	0.301***	0.432***	0.439***					0.445***	0.355***	0.428***	0.434***
					(0.093)	(0.171)	(0.094)	(0.094)					(0.093)	(0.176)	(0.094)	(0.095)
decade 1700-09					0.347***	0.219***	0.333***	0.343***					0.347***	0.274***	0.330***	0.338***
					(0.104)	(0.187)	(0.105)	(0.105)					(0.104)	(0.197)	(0.105)	(0.105)
decade 1710-19					0.355***	0.214***	0.342***	0.348***					0.353***	0.280***	0.337***	0.341***
					(0.123)	(0.210)	(0.124)	(0.125)					(0.123)	(0.223)	(0.124)	(0.125)
decade 1720-29					0.333***	0.189***	0.308***	0.322***					0.334***	0.273***	0.305***	0.318***
					(0.123)	(0.213)	(0.124)	(0.124)					(0.123)	(0.236)	(0.124)	(0.124)
decade 1730-39					0.364***	0.195***	0.324***	0.343***					0.369***	0.311***	0.325***	0.342***
					(0.122)	(0.215)	(0.124)	(0.124)					(0.122)	(0.251)	(0.124)	(0.124)
decade 1740-49					0.369***	0.183***	0.324***	0.338***					0.372***	0.321***	0.323***	0.336***
					(0.122)	(0.218)	(0.123)	(0.124)					(0.122)	(0.268)	(0.123)	(0.124)
Birth rank									0.983***	0.963***	0.985***	0.984***	0.983***	0.962***	0.985***	0.984***
									(0.003)	(0.006)	(0.003)	(0.003)	(0.003)	(0.008)	(0.003)	(0.003)
Total # of siblings									0.992*	1.003	0.991**	0.992*	0.992*	1.006	0.990**	0.992*
									(0.003)	(0.016)	(0.003)	(0.003)	(0.003)	(0.016)	(0.003)	(0.003)
# of sisters alive (at age 14)									0.999	1.031	0.997	1	1.002	1.033	0.997	1

	I	I sfe	I pfe	I bfe	II	II sfe	II pfe	II bfe	III	III sfe	III pfe	III bfe	IV	IV sfe	IV pfe	IV bfe
									(0.006)	(0.021)	(0.006)	(0.006)	(0.006)	(0.021)	(0.006)	(0.006)
# of brothers alive (at age 14)									1.008	0.933+	0.992	0.993	1.006	0.932+	0.992	0.991
									(0.008)	(0.036)	(0.009)	(0.009)	(0.009)	(0.036)	(0.009)	(0.009)
Mother alive									ref.	ref.	ref.	ref.	ref.	ref.	ref.	ref.
Mother dead									1.012	1.290***	1.009	1.008	1.018	1.291***	1.013	1.013
									(0.020)	(0.065)	(0.021)	(0.021)	(0.020)	(0.065)	(0.021)	(0.021)
Father alive									ref.	ref.	ref.	ref.	ref.	ref.	ref.	ref.
Father dead									1.105***	1.258***	1.117***	1.118***	1.110***	1.263***	1.119***	1.120***
									(0.019)	(0.047)	(0.019)	(0.019)	(0.019)	(0.047)	(0.019)	(0.019)
Num.Obs.	185810	185709	185810	185810	185810	185709	185810	185810	185472	185472	185472	185472	185472	185472	185472	185472
AIC	286128.0	23500.5	162589.0	172432.8	286000.9	23434.0	162466.4	172318.6	285321.5	23409.8	161994.3	171841.0	285190.6	23359.8	161870.9	171723.7
BIC	286143.3	23515.9	162604.4	172448.1	286069.9	23502.9	162535.4	172387.6	285382.8	23471.1	162055.6	171902.4	285305.6	23474.7	161985.8	171838.6
Log.Lik.	-143061.991	-11748.275	-81292.518	-86214.402	-142991.475	-11707.983	-81224.221	-86150.310	-142652.728	-11696.917	-80989.157	-85912.525	-142580.306	-11664.885	-80920.433	-85846.834
concordance	0.554	0.520	0.568	0.568	0.559	0.536	0.577	0.578	0.572	0.558	0.592	0.587	0.573	0.558	0.595	0.591
n	185810	185709	185810	185810	185810	185709	185810	185810	185472	185472	185472	185472	185472	185472	185472	185472
nevent	15765	15752	15765	15765	15765	15752	15765	15765	15729	15729	15729	15729	15729	15729	15729	15729
p.value.log	0	0	0	0	0	0	0	0	0	0	0	0	0	0	0	0
p.value.sc	0	0	0	0	0	0	0	0	0	0	0	0	0	0	0	0
p.value.wald	0	0	0	0	0	0	0	0	0	0	0	0	0	0	0	0
r.squared.max	0.786	0.119	0.585	0.606	0.786	0.119	0.585	0.606	0.786	0.119	0.584	0.605	0.786	0.119	0.584	0.605
statistic.log	608.620	117.579	711.307	537.902	749.651	198.161	847.902	666.085	708.882	191.522	842.207	653.688	853.726	255.586	979.655	785.069
statistic.sc	752.848	117.749	859.963	618.577	983.829	198.430	1084.072	809.879	851.779	191.673	991.304	734.323	1087.323	255.560	1217.287	929.558
statistic.wald	721.360	115.620	821.500	599.080	922.060	192.370	1012.360	768.320	819.960	188.540	952.540	714.810	1024.990	248.150	1145.320	888.150
std.error.concordance	0.002	0.002	0.003	0.003	0.003	0.004	0.004	0.004	0.003	0.005	0.004	0.004	0.003	0.005	0.004	0.004

+ p < 0.1, * p < 0.05, ** p < 0.01, *** p < 0.001

Regional: Hazard ratios from Cox regression models for women's transition into first marriage using the regional sex ratio from prospective localization

	I	I sfe	I pfe	I bfe	II	II sfe	II pfe	II bfe	III	III sfe	III pfe	III bfe	IV	IV sfe	IV pfe	IV bfe
SR <45%	0.580	0.400	0.659	0.649	0.611	0.425	0.827	0.706	0.585	0.391	0.652	0.650	0.620	0.433	0.817	0.708
	(0.447)	(0.672)	(0.456)	(0.451)	(0.448)	(0.671)	(0.456)	(0.451)	(0.447)	(0.666)	(0.456)	(0.451)	(0.448)	(0.667)	(0.457)	(0.451)
SR 45-50%	0.497***	0.610*	0.588***	0.559***	0.515***	0.636*	0.718*	0.600***	0.500***	0.574**	0.585***	0.561***	0.522***	0.621*	0.716*	0.605***
	(0.116)	(0.204)	(0.133)	(0.122)	(0.116)	(0.205)	(0.135)	(0.123)	(0.116)	(0.205)	(0.133)	(0.122)	(0.117)	(0.205)	(0.135)	(0.122)
SR 50-55%	0.952*	1.065	0.887***	0.944*	0.959+	1.094+	1.007	0.994	0.954*	1.071	0.887***	0.947*	0.961+	1.092+	1.005	0.997
	(0.022)	(0.048)	(0.031)	(0.027)	(0.023)	(0.049)	(0.032)	(0.027)	(0.023)	(0.049)	(0.031)	(0.027)	(0.023)	(0.049)	(0.032)	(0.027)
SR 55-59%	ref.	ref.	ref.	ref.	ref.	ref.	ref.	ref.	ref.	ref.	ref.	ref.	ref.	ref.	ref.	ref.
SR 60-65%	1.228***	1.126**	1.250***	1.190***	1.083**	0.996	0.968	1.008	1.224***	1.086+	1.245***	1.189***	1.084**	0.991	0.970	1.009
	(0.023)	(0.045)	(0.025)	(0.024)	(0.027)	(0.050)	(0.032)	(0.029)	(0.023)	(0.046)	(0.025)	(0.024)	(0.027)	(0.050)	(0.032)	(0.029)
SR >65%	1.890***	1.480***	1.963***	1.872***	1.045	0.913	0.900+	0.990	1.863***	1.269**	1.937***	1.860***	1.041	0.869	0.899+	0.987
	(0.033)	(0.078)	(0.035)	(0.035)	(0.054)	(0.092)	(0.059)	(0.056)	(0.033)	(0.081)	(0.035)	(0.035)	(0.054)	(0.093)	(0.059)	(0.056)
decade 1670-79					ref.	ref.	ref.	ref.					ref.	ref.	ref.	ref.
decade 1680-89					0.850+	0.614**	0.801*	0.844+					0.853+	0.696*	0.797**	0.838*
					(0.087)	(0.162)	(0.088)	(0.088)					(0.087)	(0.164)	(0.088)	(0.088)
decade 1690-99					0.502***	0.323***	0.463***	0.508***					0.505***	0.402***	0.460***	0.504***
					(0.090)	(0.183)	(0.093)	(0.092)					(0.090)	(0.189)	(0.093)	(0.092)
decade 1700-09					0.386***	0.211***	0.333***	0.382***					0.389***	0.286***	0.331***	0.377***
					(0.095)	(0.196)	(0.099)	(0.098)					(0.095)	(0.209)	(0.099)	(0.098)
decade 1710-19					0.361***	0.174***	0.289***	0.340***					0.361***	0.259***	0.286***	0.333***
					(0.095)	(0.204)	(0.101)	(0.099)					(0.095)	(0.225)	(0.101)	(0.099)
decade 1720-29					0.349***	0.155***	0.263***	0.322***					0.352***	0.259***	0.263***	0.318***
					(0.095)	(0.209)	(0.103)	(0.099)					(0.095)	(0.242)	(0.103)	(0.100)
decade 1730-39					0.375***	0.160***	0.277***	0.338***					0.383***	0.303***	0.280***	0.338***
					(0.095)	(0.212)	(0.102)	(0.099)					(0.095)	(0.259)	(0.102)	(0.099)
decade 1740-49					0.373***	0.147***	0.276***	0.331***					0.379***	0.316***	0.278***	0.329***
					(0.094)	(0.214)	(0.101)	(0.098)					(0.094)	(0.278)	(0.101)	(0.098)
Birth rank									0.984***	0.954***	0.984***	0.985***	0.984***	0.955***	0.985***	0.985***

	I	I sfe	I pfe	I bfe	II	II sfe	II pfe	II bfe	III	III sfe	III pfe	III bfe	IV	IV sfe	IV pfe	IV bfe
									(0.003)	(0.006)	(0.003)	(0.003)	(0.003)	(0.008)	(0.003)	(0.003)
Total # of siblings									0.990**	1.009	0.990**	0.990**	0.991**	1.010	0.992*	0.992*
									(0.003)	(0.017)	(0.003)	(0.003)	(0.003)	(0.017)	(0.003)	(0.003)
# of sisters alive (at age 14)									1	1.044*	0.996	0.998	1.001	1.045*	0.995	0.996
									(0.006)	(0.021)	(0.007)	(0.007)	(0.006)	(0.021)	(0.007)	(0.007)
# of brothers alive (at age 14)									1.009	0.912*	0.993	0.996	1.009	0.913*	0.993	0.995
									(0.009)	(0.037)	(0.009)	(0.009)	(0.009)	(0.037)	(0.009)	(0.009)
Mother alive									ref.	ref.	ref.	ref.	ref.	ref.	ref.	ref.
Mother dead									1	1.289***	1.003	0.996	1.017	1.308***	1.020	1.015
									(0.021)	(0.067)	(0.021)	(0.021)	(0.021)	(0.067)	(0.021)	(0.021)
Father alive									ref.	ref.	ref.	ref.	ref.	ref.	ref.	ref.
Father dead									1.099***	1.243***	1.116***	1.111***	1.108***	1.261***	1.125***	1.121***
									(0.019)	(0.048)	(0.019)	(0.019)	(0.019)	(0.049)	(0.020)	(0.019)
Num.Obs.	175212	175121	175212	175212	175212	175121	175212	175212	174898	174898	174898	174898	174898	174898	174898	174898
AIC	270633.7	21661.5	153302.9	162311.0	270408.2	21550.1	153028.5	162080.6	269851.1	21544.9	152719.9	161739.7	269627.7	21471.4	152449.9	161508.1
BIC	270671.8	21699.6	153341.0	162349.1	270499.6	21641.4	153119.9	162171.9	269934.8	21628.7	152803.7	161823.4	269764.7	21608.5	152587.0	161645.2
Log.Lik.	-135311.853	-10825.759	-76646.439	-81150.505	-135192.100	-10763.032	-76502.228	-81028.277	-134914.530	-10761.451	-76348.972	-80858.837	-134795.836	-10717.713	-76206.974	-80736.053
concordance	0.553	0.516	0.562	0.561	0.560	0.535	0.572	0.574	0.567	0.557	0.586	0.579	0.571	0.559	0.590	0.589
n	175212	175121	175212	175212	175212	175121	175212	175212	174898	174898	174898	174898	174898	174898	174898	174898
nevent	15007	14994	15007	15007	15007	14994	15007	15007	14972	14972	14972	14972	14972	14972	14972	14972
p.value.log	0	0	0	0	0	0	0	0	0	0	0	0	0	0	0	0
p.value.sc	0	0	0	0	0	0	0	0	0	0	0	0	0	0	0	0
p.value.wald	0	0	0	0	0	0	0	0	0	0	0	0	0	0	0	0
r.squared.max	0.787	0.116	0.584	0.605	0.787	0.116	0.584	0.605	0.787	0.116	0.584	0.604	0.787	0.116	0.584	0.604
statistic.log	449.028	34.947	432.977	359.722	688.534	160.403	721.399	604.178	550.224	138.951	566.720	472.908	787.611	226.427	850.716	718.477
statistic.sc	506.904	34.759	497.576	404.654	866.346	160.534	907.522	723.499	607.484	138.246	631.780	517.816	964.993	226.118	1038.348	838.421
statistic.wald	491.730	34.460	483.930	394.010	816.450	156.080	853.300	687.630	592.100	136.970	617.760	506.940	914.560	220.250	983.750	802.430
std.error.concordance	0.003	0.003	0.004	0.003	0.003	0.005	0.004	0.004	0.003	0.006	0.004	0.004	0.003	0.006	0.004	0.004

+ p < 0.1, * p < 0.05, ** p < 0.01, *** p < 0.001

Parish-level: Hazard ratios from Cox regression models for women's transition into first marriage using the parish-level sex ratio from prospective localization

	I	I sfe	I pfe	I bfe	II	II sfe	II pfe	II bfe	III	III sfe	III pfe	III bfe	IV	IV sfe	IV pfe	IV bfe
SR <45%	0.593***	0.747	0.638***	0.618***	0.599***	0.764	0.687**	0.632***	0.590***	0.730+	0.635***	0.615***	0.597***	0.747	0.683**	0.629***
	(0.117)	(0.187)	(0.125)	(0.123)	(0.117)	(0.187)	(0.125)	(0.123)	(0.118)	(0.188)	(0.125)	(0.124)	(0.118)	(0.189)	(0.126)	(0.124)
SR 45-50%	0.786***	0.979	0.799***	0.835***	0.795***	0.993	0.871**	0.854***	0.792***	0.975	0.801***	0.841***	0.802***	0.985	0.874**	0.862**
	(0.045)	(0.078)	(0.051)	(0.048)	(0.045)	(0.079)	(0.051)	(0.048)	(0.045)	(0.079)	(0.051)	(0.048)	(0.045)	(0.079)	(0.051)	(0.048)
SR 50-55%	0.974	1.062	0.937**	0.982	0.984	1.072+	0.980	0.992	0.977	1.060	0.938**	0.985	0.987	1.074+	0.982	0.997
	(0.021)	(0.038)	(0.024)	(0.023)	(0.021)	(0.038)	(0.025)	(0.023)	(0.021)	(0.038)	(0.024)	(0.023)	(0.022)	(0.038)	(0.025)	(0.023)
SR 55-59%	ref.	ref.	ref.	ref.	ref.	ref.	ref.	ref.	ref.	ref.	ref.	ref.	ref.	ref.	ref.	ref.
SR 60-65%	1.286***	1.122**	1.232***	1.251***	1.175***	1.057	0.995	1.114***	1.281***	1.088*	1.224***	1.249***	1.175***	1.051	0.993	1.114***
	(0.022)	(0.040)	(0.024)	(0.023)	(0.024)	(0.041)	(0.027)	(0.025)	(0.022)	(0.040)	(0.024)	(0.023)	(0.024)	(0.041)	(0.027)	(0.025)
SR >65%	1.521***	1.108+	1.556***	1.486***	1.038	0.884+	0.844***	1	1.502***	1.017	1.529***	1.474***	1.031	0.861*	0.835***	0.994
	(0.030)	(0.060)	(0.033)	(0.031)	(0.038)	(0.064)	(0.044)	(0.039)	(0.031)	(0.061)	(0.033)	(0.032)	(0.038)	(0.064)	(0.044)	(0.039)
decade 1670-79					ref.	ref.	ref.	ref.					ref.	ref.	ref.	ref.
decade 1680-89					0.833*	0.606**	0.776**	0.822*					0.836*	0.691*	0.771**	0.817*
					(0.086)	(0.161)	(0.087)	(0.087)					(0.086)	(0.164)	(0.087)	(0.087)
decade 1690-99					0.484***	0.315***	0.433***	0.487***					0.486***	0.398***	0.428***	0.482***
					(0.086)	(0.178)	(0.089)	(0.087)					(0.086)	(0.185)	(0.089)	(0.088)
decade 1700-09					0.374***	0.206***	0.311***	0.371***					0.375***	0.285***	0.307***	0.366***
					(0.087)	(0.188)	(0.091)	(0.090)					(0.087)	(0.202)	(0.091)	(0.090)
decade 1710-19					0.355***	0.173***	0.274***	0.339***					0.353***	0.262***	0.269***	0.332***
					(0.087)	(0.195)	(0.092)	(0.089)					(0.087)	(0.219)	(0.092)	(0.089)
decade 1720-29					0.341***	0.153***	0.250***	0.322***					0.342***	0.261***	0.247***	0.317***
					(0.086)	(0.200)	(0.092)	(0.089)					(0.086)	(0.235)	(0.092)	(0.089)
decade 1730-39					0.372***	0.159***	0.263***	0.341***					0.378***	0.308***	0.264***	0.341***
					(0.085)	(0.203)	(0.092)	(0.088)					(0.085)	(0.253)	(0.092)	(0.088)
decade 1740-49					0.369***	0.147***	0.262***	0.332***					0.373***	0.323***	0.261***	0.330***
					(0.084)	(0.206)	(0.091)	(0.088)					(0.084)	(0.273)	(0.091)	(0.088)
Birth rank									0.984***	0.952***	0.983***	0.985***	0.984***	0.955***	0.985***	0.984***

	I	I sfe	I pfe	I bfe	II	II sfe	II pfe	II bfe	III	III sfe	III pfe	III bfe	IV	IV sfe	IV pfe	IV bfe
									(0.003)	(0.006)	(0.003)	(0.003)	(0.003)	(0.008)	(0.003)	(0.003)
Total # of siblings									0.989**	1.009	0.990**	0.989**	0.991**	1.010	0.992*	0.992*
									(0.003)	(0.017)	(0.003)	(0.003)	(0.003)	(0.017)	(0.003)	(0.003)
# of sisters alive (at age 14)									1	1.044*	0.996	0.999	1.001	1.045*	0.995	0.997
									(0.006)	(0.021)	(0.007)	(0.007)	(0.006)	(0.021)	(0.007)	(0.007)
# of brothers alive (at age 14)									1.009	0.912*	0.994	0.996	1.009	0.912*	0.993	0.995
									(0.009)	(0.037)	(0.009)	(0.009)	(0.009)	(0.037)	(0.009)	(0.009)
Mother alive									ref.	ref.	ref.	ref.	ref.	ref.	ref.	ref.
Mother dead									0.994	1.287***	0.993	0.991	1.018	1.307***	1.020	1.015
									(0.021)	(0.067)	(0.021)	(0.021)	(0.021)	(0.067)	(0.021)	(0.021)
Father alive									ref.	ref.	ref.	ref.	ref.	ref.	ref.	ref.
Father dead									1.096***	1.248***	1.106***	1.107***	1.111***	1.264***	1.125***	1.123***
									(0.019)	(0.048)	(0.019)	(0.019)	(0.019)	(0.049)	(0.020)	(0.019)
Num.Obs.	175340	175249	175340	175340	175340	175249	175340	175340	175026	175026	175026	175026	175026	175026	175026	175026
AIC	270771.5	21686.0	153469.8	162449.6	270416.3	21550.0	153015.3	162099.4	269988.0	21558.8	152887.1	161878.7	269636.5	21470.5	152434.9	161526.7
BIC	270809.5	21724.0	153507.8	162487.7	270507.7	21641.4	153106.7	162190.8	270071.8	21642.6	152970.8	161962.5	269773.6	21607.5	152571.9	161663.8
Log.Lik.	-135380.730	-10837.986	-76729.883	-81219.807	-135196.133	-10763.015	-76495.652	-81037.704	-134983.011	-10768.418	-76432.539	-80928.374	-134800.248	-10717.240	-76199.440	-80745.355
concordance	0.553	0.509	0.563	0.560	0.566	0.539	0.571	0.578	0.565	0.557	0.586	0.577	0.575	0.558	0.591	0.590
n	175340	175249	175340	175340	175340	175249	175340	175340	175026	175026	175026	175026	175026	175026	175026	175026
nevent	15010	14997	15010	15010	15010	14997	15010	15010	14975	14975	14975	14975	14975	14975	14975	14975
p.value.log	0	0.017	0	0	0	0	0	0	0	0	0	0	0	0	0	0
p.value.sc	0	0.017	0	0	0	0	0	0	0	0	0	0	0	0	0	0
p.value.wald	0	0.017	0	0	0	0	0	0	0	0	0	0	0	0	0	0
r.squared.max	0.787	0.116	0.584	0.605	0.787	0.116	0.584	0.605	0.787	0.116	0.583	0.604	0.787	0.116	0.583	0.604
statistic.log	374.048	13.842	277.932	270.916	743.243	163.783	746.395	635.122	476.015	128.365	411.432	383.560	841.541	230.720	877.629	749.597
statistic.sc	390.892	13.759	295.593	282.386	927.203	164.089	933.563	756.121	491.609	127.778	429.080	394.818	1024.572	230.458	1066.217	871.041
statistic.wald	385.450	13.730	292.860	279.630	877.220	159.500	877.770	720.190	486	126.840	426.030	391.880	973.950	224.380	1010.160	834.920
std.error.concordance	0.003	0.004	0.004	0.004	0.003	0.005	0.004	0.004	0.003	0.006	0.004	0.004	0.003	0.006	0.004	0.004

+ p < 0.1, * p < 0.05, ** p < 0.01, *** p < 0.001

4 Men, prospective localization

Colonial: Hazard ratios from Cox regression models for men's transition into first marriage using the colonial sex ratio from prospective localization

	I	I sfe	I pfe	I bfe	II	II sfe	II pfe	II bfe	III	III sfe	III pfe	III bfe	IV	IV sfe	IV pfe	IV bfe
SR 55-59%	ref.	ref.	ref.	ref.	ref.	ref.	ref.	ref.	ref.	ref.	ref.	ref.	ref.	ref.	ref.	ref.
SR 60-65%	0.893***	0.860*	0.962	0.932*	0.948	0.955	0.940	0.963	0.890***	0.870*	0.956	0.929*	0.949	0.944	0.941	0.966
	(0.028)	(0.067)	(0.029)	(0.031)	(0.075)	(0.109)	(0.076)	(0.076)	(0.029)	(0.071)	(0.030)	(0.031)	(0.075)	(0.110)	(0.076)	(0.076)
SR >65%	0.867***	0.774*	0.980	0.944	0.827	0.766	0.838	0.842	0.868***	0.785*	0.981	0.947	0.829	0.744+	0.841	0.844
	(0.042)	(0.112)	(0.044)	(0.045)	(0.117)	(0.174)	(0.118)	(0.118)	(0.042)	(0.122)	(0.044)	(0.046)	(0.117)	(0.177)	(0.118)	(0.118)
decade 1670-79					ref.	ref.	ref.	ref.					ref.	ref.	ref.	ref.
decade 1680-89					1.433**	1.570*	1.421**	1.406**					1.433**	1.623*	1.420**	1.403**
					(0.127)	(0.204)	(0.127)	(0.128)					(0.127)	(0.205)	(0.127)	(0.128)
decade 1690-99					1.420*	1.435	1.401*	1.319+					1.416*	1.513+	1.392*	1.309+
					(0.141)	(0.234)	(0.141)	(0.142)					(0.141)	(0.238)	(0.141)	(0.142)
decade 1700-09					1.088	1.163	1.033	1.005					1.081	1.247	1.024	0.992
					(0.150)	(0.250)	(0.151)	(0.152)					(0.151)	(0.259)	(0.151)	(0.152)
decade 1710-19					1.266	1.415	1.191	1.184					1.263	1.536	1.188	1.175
					(0.166)	(0.272)	(0.167)	(0.168)					(0.166)	(0.284)	(0.167)	(0.168)
decade 1720-29					1.292	1.531	1.176	1.179					1.291	1.707+	1.176	1.172
					(0.166)	(0.275)	(0.167)	(0.167)					(0.166)	(0.297)	(0.167)	(0.168)
decade 1730-39					1.312	1.557	1.142	1.177					1.313	1.782+	1.141	1.170
					(0.165)	(0.278)	(0.167)	(0.167)					(0.166)	(0.311)	(0.167)	(0.167)
decade 1740-49					1.307	1.562	1.116	1.146					1.310	1.844+	1.115	1.141
					(0.165)	(0.280)	(0.167)	(0.167)					(0.165)	(0.329)	(0.167)	(0.167)
Birth rank									1.007+	0.993	1.006+	1.006	1.007*	0.984+	1.007*	1.006+
									(0.003)	(0.007)	(0.003)	(0.003)	(0.003)	(0.009)	(0.003)	(0.003)
Total # of siblings									0.987***	0.990	0.988**	0.989**	0.986***	0.992	0.988**	0.989**
									(0.004)	(0.019)	(0.004)	(0.004)	(0.004)	(0.019)	(0.004)	(0.004)
# of sisters alive (at age 14)									0.998	0.975	0.989	0.993	0.999	0.979	0.988	0.992

	I	I sfe	I pfe	I bfe	II	II sfe	II pfe	II bfe	III	III sfe	III pfe	III bfe	IV	IV sfe	IV pfe	IV bfe
									(0.007)	(0.032)	(0.007)	(0.007)	(0.007)	(0.032)	(0.007)	(0.007)
# of brothers alive (at age 14)									1.018+	1.003	1.008	1.005	1.017+	0.998	1.008	1.005
									(0.009)	(0.040)	(0.009)	(0.009)	(0.009)	(0.040)	(0.009)	(0.009)
Mother alive									ref.	ref.	ref.	ref.	ref.	ref.	ref.	ref.
Mother dead									1.029	1.078	1.024	1.019	1.032	1.071	1.026	1.022
									(0.022)	(0.070)	(0.022)	(0.022)	(0.022)	(0.071)	(0.022)	(0.022)
Father alive									ref.	ref.	ref.	ref.	ref.	ref.	ref.	ref.
Father dead									0.997	1.054	1.004	1	1.002	1.065	1.006	1.003
									(0.020)	(0.051)	(0.020)	(0.020)	(0.020)	(0.052)	(0.020)	(0.020)
Num.Obs.	231097	230953	231097	231097	231097	230953	231097	231097	230816	230816	230816	230816	230816	230816	230816	230816
AIC	219011.4	17605.2	118784.7	130083.1	218982.8	17600.4	118750.3	130057.9	218722.9	17597.7	118592.0	129869.6	218692.9	17591.7	118557.1	129843.3
BIC	219026.2	17620.1	118799.5	130098.0	219049.7	17667.3	118817.2	130124.7	218782.3	17657.1	118651.3	129929.0	218804.3	17703.1	118668.5	129954.7
Log.Lik.	-109503.691	-8800.607	-59390.331	-65039.559	-109482.413	-8791.222	-59366.172	-65019.950	-109353.431	-8790.828	-59287.975	-64926.797	-109331.470	-8780.869	-59263.543	-64906.670
concordance	0.509	0.506	0.505	0.508	0.514	0.516	0.520	0.515	0.522	0.519	0.520	0.523	0.526	0.528	0.529	0.529
n	231097	230953	231097	231097	231097	230953	231097	231097	230816	230816	230816	230816	230816	230816	230816	230816
nevent	12403	12396	12403	12403	12403	12396	12403	12403	12389	12389	12389	12389	12389	12389	12389	12389
p.value.log	0	0.035	0.404	0.045	0	0.003	0	0	0	0.122	0	0	0	0.005	0	0
p.value.sc	0	0.035	0.407	0.046	0	0.003	0	0	0	0.120	0	0	0	0.006	0	0
p.value.wald	0	0.036	0.407	0.046	0	0.003	0	0	0	0.121	0	0	0	0.006	0	0
r.squared.max	0.612	0.073	0.402	0.430	0.612	0.073	0.402	0.430	0.612	0.073	0.402	0.430	0.612	0.073	0.402	0.430
statistic.log	25.943	6.678	1.810	6.214	68.499	25.448	50.129	45.432	47.891	12.711	37.876	32.616	91.813	32.630	86.741	72.871
statistic.sc	25.250	6.677	1.797	6.152	64.135	25.142	49.027	43.704	47.119	12.762	37.722	32.474	87.339	32.419	85.471	71.031
statistic.wald	25.220	6.670	1.800	6.150	63.710	25	48.840	43.520	47.080	12.740	37.710	32.470	86.900	32.230	85.270	70.840
std.error.concordance	0.002	0.002	0.003	0.003	0.003	0.005	0.004	0.004	0.003	0.006	0.004	0.004	0.003	0.006	0.004	0.004

+ p < 0.1, * p < 0.05, ** p < 0.01, *** p < 0.001

Regional: Hazard ratios from Cox regression models for men's transition into first marriage using the regional sex ratio from prospective localization

	I	I sfe	I pfe	I bfe	II	II sfe	II pfe	II bfe	III	III sfe	III pfe	III bfe	IV	IV sfe	IV pfe	IV bfe
SR <45%	1.007	0.207	1.605	1.108	1	0.197	1.632	1.091	1.008	0.211	1.605	1.109	1.002	0.198	1.624	1.091
	(0.408)	(1.088)	(0.422)	(0.412)	(0.409)	(1.089)	(0.423)	(0.413)	(0.409)	(1.087)	(0.422)	(0.412)	(0.409)	(1.089)	(0.423)	(0.413)
SR 45-50%	0.647***	1.015	0.922	0.776+	0.645***	0.978	0.940	0.767+	0.648***	1.019	0.923	0.772+	0.647***	0.983	0.938	0.764*
	(0.131)	(0.254)	(0.151)	(0.136)	(0.131)	(0.255)	(0.153)	(0.137)	(0.131)	(0.254)	(0.151)	(0.136)	(0.131)	(0.255)	(0.153)	(0.137)
SR 50-55%	1.090***	0.979	0.995	1.040	1.081**	0.962	1.021	1.039	1.088***	0.978	0.997	1.042	1.079**	0.963	1.022	1.041
	(0.025)	(0.049)	(0.034)	(0.028)	(0.025)	(0.050)	(0.035)	(0.029)	(0.025)	(0.049)	(0.034)	(0.028)	(0.025)	(0.050)	(0.035)	(0.029)
SR 55-59%	ref.	ref.	ref.	ref.	ref.	ref.	ref.	ref.	ref.	ref.	ref.	ref.	ref.	ref.	ref.	ref.
SR 60-65%	0.968	0.858**	0.949+	0.953+	0.969	0.858**	0.881***	0.936*	0.967	0.862**	0.948+	0.954+	0.969	0.858**	0.882***	0.937*
	(0.025)	(0.048)	(0.028)	(0.027)	(0.029)	(0.051)	(0.035)	(0.031)	(0.025)	(0.048)	(0.028)	(0.027)	(0.029)	(0.051)	(0.035)	(0.031)
SR >65%	0.786***	0.628***	0.841***	0.861**	0.751***	0.605***	0.674***	0.775***	0.785***	0.635***	0.841***	0.864**	0.749***	0.601***	0.672***	0.774***
	(0.044)	(0.095)	(0.046)	(0.046)	(0.066)	(0.111)	(0.072)	(0.067)	(0.044)	(0.098)	(0.046)	(0.046)	(0.066)	(0.111)	(0.072)	(0.067)
decade 1670-79					ref.	ref.	ref.	ref.					ref.	ref.	ref.	ref.
decade 1680-89					1.800***	2.178**	1.777***	1.768***					1.802***	2.232**	1.771***	1.761***
					(0.153)	(0.257)	(0.154)	(0.154)					(0.153)	(0.258)	(0.154)	(0.154)
decade 1690-99					1.739***	2.034**	1.642**	1.680***					1.730***	2.143**	1.621**	1.661**
					(0.155)	(0.274)	(0.157)	(0.156)					(0.155)	(0.279)	(0.157)	(0.156)
decade 1700-09					1.295	1.555	1.126	1.232					1.283	1.671+	1.108	1.210
					(0.159)	(0.287)	(0.162)	(0.161)					(0.159)	(0.298)	(0.162)	(0.161)
decade 1710-19					1.509**	1.953*	1.263	1.418*					1.498*	2.137*	1.248	1.397*
					(0.159)	(0.294)	(0.164)	(0.161)					(0.159)	(0.312)	(0.164)	(0.161)
decade 1720-29					1.553**	2.081*	1.243	1.422*					1.546**	2.334**	1.231	1.403*
					(0.159)	(0.299)	(0.165)	(0.161)					(0.159)	(0.328)	(0.165)	(0.162)
decade 1730-39					1.575**	2.083*	1.214	1.419*					1.569**	2.394*	1.201	1.400*
					(0.158)	(0.301)	(0.165)	(0.161)					(0.158)	(0.344)	(0.165)	(0.161)
decade 1740-49					1.561**	2.144*	1.196	1.377*					1.558**	2.537*	1.183	1.360+
					(0.158)	(0.303)	(0.164)	(0.161)					(0.158)	(0.362)	(0.164)	(0.161)
Birth rank									1.007*	0.995	1.006+	1.007+	1.007*	0.986	1.007*	1.007*

	I	I sfe	I pfe	I bfe	II	II sfe	II pfe	II bfe	III	III sfe	III pfe	III bfe	IV	IV sfe	IV pfe	IV bfe
									(0.003)	(0.007)	(0.004)	(0.003)	(0.003)	(0.010)	(0.004)	(0.003)
Total # of siblings									0.986***	0.987	0.988**	0.988**	0.985***	0.988	0.988**	0.988**
									(0.004)	(0.019)	(0.004)	(0.004)	(0.004)	(0.019)	(0.004)	(0.004)
# of sisters alive (at age 14)									0.997	0.980	0.987+	0.990	0.999	0.984	0.988+	0.991
									(0.007)	(0.033)	(0.007)	(0.007)	(0.007)	(0.034)	(0.007)	(0.007)
# of brothers alive (at age 14)									1.020*	1.004	1.009	1.009	1.019*	0.999	1.008	1.008
									(0.009)	(0.041)	(0.010)	(0.010)	(0.009)	(0.041)	(0.010)	(0.010)
Mother alive									ref.	ref.	ref.	ref.	ref.	ref.	ref.	ref.
Mother dead									1.031	1.077	1.025	1.022	1.034	1.065	1.030	1.026
									(0.022)	(0.073)	(0.022)	(0.022)	(0.022)	(0.074)	(0.022)	(0.022)
Father alive									ref.	ref.	ref.	ref.	ref.	ref.	ref.	ref.
Father dead									0.992	1.064	1.002	0.997	0.997	1.068	1.006	1.001
									(0.020)	(0.053)	(0.021)	(0.021)	(0.020)	(0.054)	(0.021)	(0.021)
Num.Obs.	215531	215456	215531	215531	215531	215456	215531	215531	215365	215365	215365	215365	215365	215365	215365	215365
AIC	206711.4	15976.9	111402.6	122159.5	206665.6	15961.1	111342.0	122118.8	206471.0	15971.9	111243.2	121983.9	206423.1	15955.9	111181.6	121941.8
BIC	206748.3	16013.8	111439.5	122196.4	206754.1	16049.6	111430.5	122207.3	206552.1	16053.1	111324.3	122065.0	206555.8	16088.7	111314.3	122074.5
Log.Lik.	-103350.712	-7983.456	-55696.321	-61074.759	-103320.822	-7968.567	-55658.987	-61047.384	-103224.489	-7974.971	-55610.580	-60980.935	-103193.542	-7959.960	-55572.783	-60952.880
concordance	0.518	0.514	0.510	0.516	0.527	0.524	0.524	0.531	0.527	0.525	0.522	0.528	0.534	0.538	0.532	0.536
n	215531	215456	215531	215531	215531	215456	215531	215531	215365	215365	215365	215365	215365	215365	215365	215365
nevent	11795	11789	11795	11795	11795	11789	11795	11795	11783	11783	11783	11783	11783	11783	11783	11783
p.value.log	0	0	0.003	0.001	0	0	0	0	0	0	0	0	0	0	0	0
p.value.sc	0	0	0.003	0.002	0	0	0	0	0	0	0	0	0	0	0	0
p.value.wald	0	0	0.003	0.002	0	0	0	0	0	0	0	0	0	0	0	0
r.squared.max	0.617	0.072	0.404	0.433	0.617	0.072	0.404	0.433	0.617	0.072	0.404	0.433	0.617	0.072	0.404	0.433
statistic.log	65.150	29.875	18.382	19.978	124.930	59.653	93.049	74.727	89.237	35.520	55.297	50.014	151.132	65.542	130.890	106.125
statistic.sc	61.725	29.221	18.070	19.466	114.504	57.694	89.273	70.486	85.660	34.933	54.791	49.372	140.605	63.744	127.040	101.773
statistic.wald	61.310	28.650	18.010	19.430	112.910	56.210	88.570	69.880	85.250	34.310	54.720	49.330	139	62.150	126.310	101.150
std.error.concordance	0.003	0.004	0.003	0.004	0.003	0.006	0.004	0.004	0.003	0.006	0.004	0.004	0.003	0.006	0.005	0.004

+ p < 0.1, * p < 0.05, ** p < 0.01, *** p < 0.001

Parish-level: Hazard ratios from Cox regression models for men's transition into first marriage using the parish-level sex ratio from prospective localization

	I	I sfe	I pfe	I bfe	II	II sfe	II pfe	II bfe	III	III sfe	III pfe	III bfe	IV	IV sfe	IV pfe	IV bfe
SR <45%	0.781	0.999	0.920	0.808	0.774	0.993	0.932	0.802	0.783	0.992	0.918	0.804	0.776	0.982	0.928	0.798
	(0.161)	(0.271)	(0.179)	(0.166)	(0.161)	(0.271)	(0.180)	(0.166)	(0.161)	(0.270)	(0.179)	(0.166)	(0.161)	(0.271)	(0.179)	(0.166)
SR 45-50%	1.122*	1.011	1.140*	1.140*	1.115*	0.999	1.165*	1.131*	1.120*	1.009	1.135*	1.139*	1.112*	0.998	1.158*	1.130*
	(0.053)	(0.093)	(0.060)	(0.055)	(0.053)	(0.093)	(0.060)	(0.055)	(0.053)	(0.093)	(0.060)	(0.055)	(0.053)	(0.093)	(0.060)	(0.055)
SR 50-55%	1.112***	1.073+	1.049+	1.094***	1.107***	1.061	1.060*	1.090**	1.109***	1.070	1.047	1.094***	1.104***	1.058	1.057+	1.090**
	(0.025)	(0.042)	(0.028)	(0.026)	(0.025)	(0.042)	(0.028)	(0.026)	(0.025)	(0.042)	(0.028)	(0.026)	(0.025)	(0.042)	(0.028)	(0.026)
SR 55-59%	ref.	ref.	ref.	ref.	ref.	ref.	ref.	ref.	ref.	ref.	ref.	ref.	ref.	ref.	ref.	ref.
SR 60-65%	1.025	0.979	0.938*	1.008	1.033	0.984	0.876***	1	1.025	0.981	0.940*	1.011	1.033	0.983	0.879***	1.003
	(0.024)	(0.042)	(0.027)	(0.025)	(0.025)	(0.042)	(0.029)	(0.026)	(0.024)	(0.042)	(0.027)	(0.025)	(0.026)	(0.042)	(0.029)	(0.026)
SR >65%	0.796***	0.705***	0.768***	0.833***	0.792***	0.705***	0.640***	0.795***	0.797***	0.708***	0.767***	0.837***	0.792***	0.701***	0.641***	0.799***
	(0.035)	(0.063)	(0.038)	(0.036)	(0.041)	(0.066)	(0.048)	(0.042)	(0.035)	(0.063)	(0.038)	(0.036)	(0.041)	(0.066)	(0.048)	(0.042)
decade 1670-79					ref.	ref.	ref.	ref.					ref.	ref.	ref.	ref.
decade 1680-89					1.815***	2.253**	1.769***	1.766***					1.818***	2.312**	1.765***	1.760***
					(0.153)	(0.256)	(0.154)	(0.154)					(0.153)	(0.257)	(0.154)	(0.154)
decade 1690-99					1.794***	2.194**	1.629**	1.685***					1.790***	2.321**	1.613**	1.672***
					(0.150)	(0.266)	(0.151)	(0.151)					(0.150)	(0.272)	(0.151)	(0.151)
decade 1700-09					1.357*	1.766*	1.109	1.253					1.349*	1.907*	1.095	1.237
					(0.150)	(0.276)	(0.153)	(0.152)					(0.150)	(0.288)	(0.153)	(0.152)
decade 1710-19					1.587**	2.259**	1.221	1.450*					1.581**	2.484**	1.212	1.437*
					(0.150)	(0.281)	(0.153)	(0.152)					(0.150)	(0.302)	(0.153)	(0.152)
decade 1720-29					1.629**	2.410**	1.191	1.451*					1.626**	2.717**	1.185	1.441*
					(0.149)	(0.286)	(0.153)	(0.151)					(0.149)	(0.318)	(0.153)	(0.151)
decade 1730-39					1.674***	2.426**	1.157	1.461*					1.672***	2.805**	1.150	1.450*
					(0.149)	(0.289)	(0.153)	(0.151)					(0.149)	(0.335)	(0.153)	(0.151)
decade 1740-49					1.664***	2.480**	1.132	1.417*					1.666***	2.953**	1.126	1.408*
					(0.148)	(0.291)	(0.153)	(0.151)					(0.148)	(0.354)	(0.153)	(0.151)
Birth rank									1.007*	0.996	1.006	1.006+	1.007*	0.985	1.007*	1.007+

	I	I sfe	I pfe	I bfe	II	II sfe	II pfe	II bfe	III	III sfe	III pfe	III bfe	IV	IV sfe	IV pfe	IV bfe
									(0.003)	(0.007)	(0.004)	(0.003)	(0.003)	(0.010)	(0.004)	(0.003)
Total # of siblings									0.987***	0.989	0.988**	0.988**	0.985***	0.990	0.989**	0.988**
									(0.004)	(0.019)	(0.004)	(0.004)	(0.004)	(0.019)	(0.004)	(0.004)
# of sisters alive (at age 14)									0.998	0.978	0.988	0.991	0.999	0.982	0.988	0.991
									(0.007)	(0.033)	(0.007)	(0.007)	(0.007)	(0.034)	(0.007)	(0.007)
# of brothers alive (at age 14)									1.020*	1.006	1.009	1.008	1.019*	1.002	1.008	1.008
									(0.009)	(0.041)	(0.010)	(0.010)	(0.009)	(0.041)	(0.010)	(0.010)
Mother alive									ref.	ref.	ref.	ref.	ref.	ref.	ref.	ref.
Mother dead									1.033	1.084	1.021	1.022	1.035	1.070	1.029	1.027
									(0.022)	(0.073)	(0.022)	(0.022)	(0.022)	(0.073)	(0.022)	(0.022)
Father alive									ref.	ref.	ref.	ref.	ref.	ref.	ref.	ref.
Father dead									0.994	1.071	1	0.997	0.999	1.074	1.006	1.002
									(0.020)	(0.053)	(0.021)	(0.021)	(0.020)	(0.054)	(0.021)	(0.021)
Num.Obs.	215794	215719	215794	215794	215794	215719	215794	215794	215628	215628	215628	215628	215628	215628	215628	215628
AIC	206768.1	15971.1	111359.4	122181.4	206721.1	15955.9	111278.6	122141.8	206529.1	15965.4	111200.9	122007.6	206479.9	15950.4	111120.5	121966.8
BIC	206805.0	16008.0	111396.3	122218.3	206809.6	16044.4	111367.1	122230.3	206610.2	16046.6	111282.0	122088.7	206612.7	16083.2	111253.3	122099.5
Log.Lik.	-103379.060	-7980.554	-55674.694	-61085.692	-103348.559	-7965.962	-55627.286	-61058.881	-103253.542	-7971.718	-55589.462	-60992.814	-103221.952	-7957.224	-55542.255	-60965.390
concordance	0.523	0.518	0.515	0.528	0.533	0.527	0.530	0.537	0.530	0.529	0.525	0.534	0.537	0.538	0.536	0.542
n	215794	215719	215794	215794	215794	215719	215794	215794	215628	215628	215628	215628	215628	215628	215628	215628
nevent	11799	11793	11799	11799	11799	11793	11799	11799	11787	11787	11787	11787	11787	11787	11787	11787
p.value.log	0	0	0	0	0	0	0	0	0	0	0	0	0	0	0	0
p.value.sc	0	0	0	0	0	0	0	0	0	0	0	0	0	0	0	0
p.value.wald	0	0	0	0	0	0	0	0	0	0	0	0	0	0	0	0
r.squared.max	0.617	0.071	0.403	0.432	0.617	0.071	0.403	0.432	0.616	0.071	0.403	0.432	0.616	0.071	0.403	0.432
statistic.log	87.447	39.839	68.695	53.843	148.449	69.023	163.511	107.465	110.114	46.184	104.591	81.966	173.293	75.172	199.005	136.814
statistic.sc	84.059	39.035	65.808	52.482	138.086	66.922	156.988	102.164	106.615	45.441	101.587	80.497	162.872	73.194	192.310	131.378
statistic.wald	83.620	38.750	65.560	52.330	136.470	65.720	156.260	101.490	106.170	45.110	101.320	80.330	161.240	71.880	191.550	130.690
std.error.concordance	0.003	0.005	0.004	0.004	0.003	0.006	0.004	0.004	0.003	0.006	0.004	0.004	0.003	0.006	0.005	0.004

+ p < 0.1, * p < 0.05, ** p < 0.01, *** p < 0.001
