# Supplementary material for: Sex ratios and union formation in the historical population of the St. Lawrence Valley
Source: PLoS One. 2022 Jun 8;17(6):e0268039. doi: 10.1371/journal.pone.0268039 (PMC9176776; doi:10.1371/journal.pone.0268039)

1 Supplement

Figure S1: Mean sex ratio by parish age. Parish-level SR estimates for unmarried individuals by localization method and different parish age-cutoffs

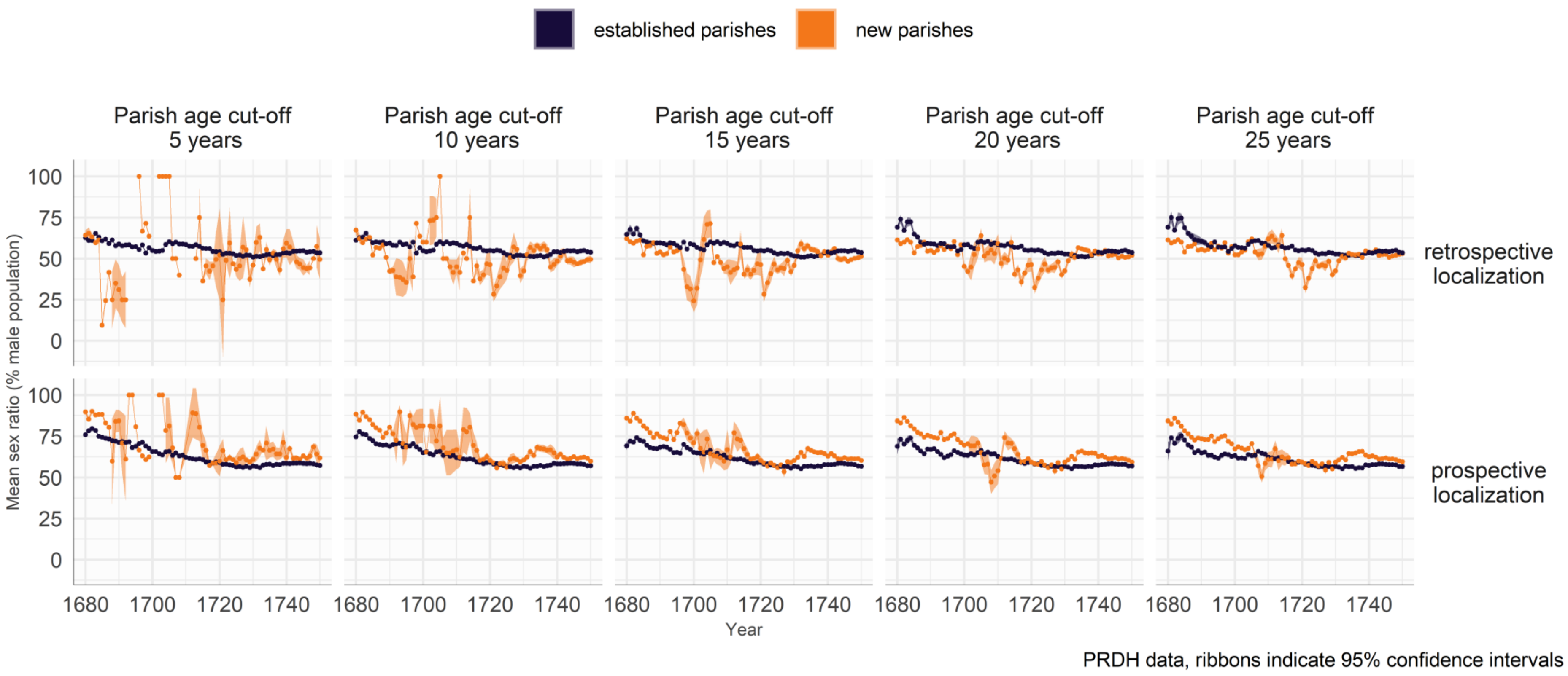

Supplement: S1 Fig — Parish-level SR estimates for unmarried individuals by localization method and different parish age cut-offs. (PDF) [file pone.0268039.s002.pdf]
