# Supplementary material for: Sex ratios and union formation in the historical population of the St. Lawrence Valley
Source: PLoS One. 2022 Jun 8;17(6):e0268039. doi: 10.1371/journal.pone.0268039 (PMC9176776; doi:10.1371/journal.pone.0268039)
Supplement: Attachment — Submitted filename: PlosOne Review.pdf [file pone.0268039.s003.pdf]

Referee Report for "Sex ratios and union formation in the historical population of the St. Lawrence Valley"

PlosOne

Jan 2022

1 Summary and main suggestions

First, let me apologize profusely for the delay in sending in this review. Things have been somewhat intense and when it rains, it pours. This does not excuse my lateness however and I wish to apologize to you for the delay.

Now, let me state that I am a weird kind of reviewer. I often read interesting papers and I wish they had been written in a different manner to better convey the point. However, I try to limit this reflex of mine as it seems pretentious and arrogant to propose to someone to rewrite a paper based on my subjective preferences. Consequently, I always try to move to the core of the argument and I only propose restructuring when absolutely crucial. In the present case, I felt no need to exercise this reflex. The paper is clear. More importantly, it makes a valuable *empirical* contribution. However, the paper is weaker with regards to the economic history and some minor modifications are warranted.

The main claim of the paper is that Quebec, with its high-quality demographic data (a well-known fact that the authors exploit), is well-suited to settle empirical conflicts regarding partnering behavior that stem *only* from data quality. I believe that this does reduce the uncertainty in the field to a narrower set (which is good) that includes environmental and institutional factors. As such, for that reason alone, the paper is worthy of publication.

1.1 Economic history

The authors are, however, a bit careless in their historical details. We know that most males who migrated came as soldiers who were subsidized for the transatlantic travel and were offered incentives to stay behind after fighting the Iroquois (this is known as the Carignan-Sallieres regiment). However, the authors omit important sources of *male* migration – indentured servants, deported salt smugglers, wayward sons given *lettres de cachet* by the crown (Paul, 2008). Overall, some 30,000 people came to New France – but only 15,000 remained

(Boleda, 1990). As a share of the first number, Boleda (1990) places the share at 1/3 but probably a smaller share when one considers only those who stayed.

Why is that an issue one might wonder? Because of how Moogk describes the migrants to New France: as reluctant migrants (Moogk, 1989). Reluctant because many were sent against their will in one way or another or had to be heavily subsidized to do so. Of all the migrants, only the indentured workers and a few others can be qualified as willing.Recent wage, income and price assembled evidence by Geloso (2019) and Geloso (2020) show that the inhabitants of New France were at best only modestly richer than the inhabitants of France (+20%) and at worst equally rich as the inhabitants of France. This suggest small to no gains to migration in contrast to the Spanish and British colonies where income gains for settlers were in excess of 2:1 relative to the respective mother countries.[1]

This causes a (minor) problem of interpretation because those who were deported/exiled/chosen by the government might have been selected on the basis of a factor that could be correlated with fertility decisions. For example, Galenson (1981) (for the American colonies), finds that a greater proportion of indentured servants migration meant more women coming of their own will. Indeed, the skewed sex ratio would entail a premium for women which would eventually reflect itself in the terms offered in indenture contracts. This premium would entice migration. In New France, a small share of indenture servitude would have hindered that mechanism.

Now, notice I said it was a minor problem. That is because that concern does not alter (I do not see how) the results of the authors. However, it does speak to how they could extend their article to speak to two other literature: a) economic history; b) economics of immigration. I think that a small tweak to incorporate the elements above might allow the article to have a greater reach. Indeed, it may also even speak to points made by Gillian Hamilton on why Quebec had strange (and exceptionally strong) property rights regimes for women in marriage (compared to the rest of the Americas) (Hamilton, 1999).

1.2 Age Gap Across Gender At Marriage

I would also encourage the authors to try and provide contemporary comparison on the age gap at marriage. For example, it would be good to know what was the gap in France and the American colonies to the south of Quebec. I would recommend the works of Diebolt et al. (2017) on France as it contains a ton of statistical information for France from 1740 onwards. I would be satisfied with a comparison in 1740 for example. All I think is necessary is a comparison to highlight that Quebec was indeed exceptional.

[1]Some evidence for the Saint-Domingue colony suggest that French settlers also stood to gain much from going there instead of New France which is why Eltis (1999) estimates that 90% of all net migration out of France went to Saint-Domingue and not the Canadian holdings.

1.3 A short question

The authors claim that "Therefore, our findings do not support concerns that male-skewed sex ratio contexts necessarily result in an increase of never married men". I would be careful here as there is a question that lingers about male mortality. Many males were involved seasonally in the fur trade – a physically demanding but economically remunerative trade. If men died in sufficient numbers during altercations with the natives or as deaths during the trade, the results would be driven by this would it not? Moreover, men had militia duty of importance to fight off the natives. This meant also greater mortality. As such, the results could be driven by the overall level of violence as well? Is that a correct claim? Should it be added to the section on page 25?

References

Boleda, M. (1990). Trente mille français à la conquête du saint-laurent. *Histoire sociale/Social History.*

Diebolt, C., Perrin, F., et al. (2017). *Understanding Demographic Transitions.* Springer.

Eltis, D. (1999). Atlantic history in global perspective. *Itinerario*, 23(2):141–161.

Galenson, D. W. (1981). The market evaluation of human capital: The case of indentured servitude. *Journal of Political Economy*, 89(3):446–467.

Geloso, V. (2020). Predation, seigneurial tenure, and development in french colonial america. *Social Science History*, 44(4):747–770.

Geloso, V. J. (2019). Distinct within north america: living standards in french canada, 1688–1775. *Cliometrica*, 13(2):277–321.

Hamilton, G. (1999). Property rights and transaction costs in marriage: Evidence from prenuptial contracts. *The Journal of Economic History*, 59(1):68–103.

Moogk, P. N. (1989). Reluctant exiles: Emigrants from france in canada before 1760. *The William and Mary Quarterly: A Magazine of Early American History and*, pages 463–505.

Paul, J. (2008). *Exilés au nom du roi: Les fils de famille et les faux-sauniers en Nouvelle-France. 1723-1749.* Septentrion.
